# East African City Centers Show Lower PM_2.5_ Levels than Their Suburbs

**DOI:** 10.1021/acs.estlett.5c00451

**Published:** 2025-06-09

**Authors:** Samuel De Xun Chua, Otienoh Oguge, Celestine Atieno Oliewo, Richard Sserunjogi, Deo Okure, Priscilla Adong, Asinta Manyele, Tareq Hussein, Yuheng Yang, Xixi Lu, Katrianne Lehtipalo, Martha Arbayani Zaidan, Tuukka Petäjä

**Affiliations:** † Institute for Atmospheric and Earth System Research, Faculty of Science, 111628 University of Helsinki, Helsinki, 00014, Finland; ‡ Department of Geography, 37580 National University of Singapore, Singapore, 119077, Singapore; § Eastern Africa GEOHealth Hub, Centre for Advanced Studies in Environmental Law and Policy, Faculty of Law, 309376 University of Nairobi, Nairobi, 00100, Kenya; ∥ “Enzo Ferrari” Department of Engineering, 9306 University of Modena and Reggio Emilia, Modena, 41121, Italy; ⊥ AirQo, Department of Computer Science, 58588 Makerere University, Kampala, 7062, Uganda; # Electronics and Telecommunication Engineering, 127799 Dar es Salaam Institute of Technology, Dar es Salaam, 2958, Tanzania; ∇ Finnish Meteorological Institute, Helsinki, 00101, Finland; ○ Department of Computer Science, Faculty of Science, 111628 University of Helsinki, Helsinki, 00014, Finland

**Keywords:** Air pollution, Urban air quality, African cities, Remote sensing, Low-cost sensors

## Abstract

Urban air pollution remains a pressing challenge in rapidly developing economies, particularly in data-scarce regions. This study examined air quality in three major East African cities—Kampala, Nairobi, and Dar es Salaam—by integrating low-cost air sensors with satellite data to produce 1 km × 1 km resolution daily PM_2.5_ (particulate matter smaller than 2.5 μm) maps from 2019 to 2023. Average PM_2.5_ concentrations were 31.4 ± 6.6 μg/m^3^ around Kampala, 21.7 ± 2.8 μg/m^3^ around Nairobi, and 33.1 ± 7.4 μg/m^3^ around Dar es Salaam, indicating moderate to unhealthy levels of air quality. Unexpectedly, urban centers exhibited lower PM_2.5_ levels than surrounding suburban area. This discrepancy is likely due to combustion-related activities that occur in the suburbs. Such results suggest that air quality mitigation efforts must extend beyond urban centers to suburban areas, where seasonal vegetation loss and combustion processes may drive pollution spikes. Beyond presenting a scalable approach for monitoring air quality in data-scarce regions, this study highlights the importance of localized strategies for urban air quality management.

## Graphical abstract

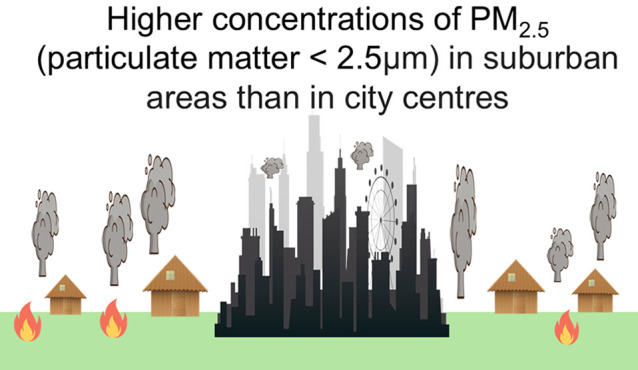

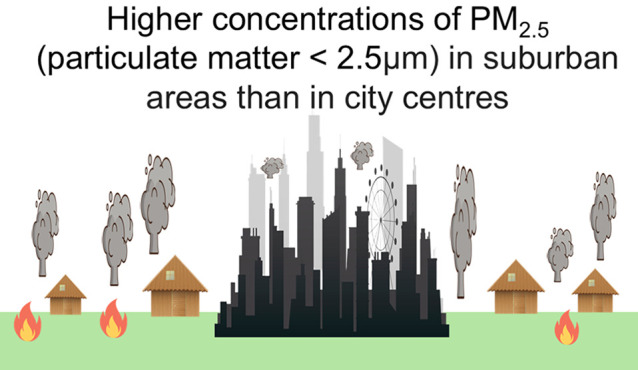

## Introduction

As global urbanization rates rise, ensuring that cities are “safe, resilient, and sustainable”—a key target highlighted by the UN’s Sustainable Development Goals—has become a primary focus for urban planners. Particularly, air pollution remains a persistent issue for many cities,^1–3^ with studies establishing clear linkages between polluted air and adverse health outcomes,^4^ increased poverty rates^5^ and impaired child development.^4,6^ This issue is particularly acute for urban communities in emerging economies, where rapid urban growth is expected to increase population vulnerability there in the near future.^7^

Before effective action can be taken to mitigate air pollution, accurate and extensive monitoring systems are essential for identifying and responding to poor air quality. Thus, particulate matter smaller than 2.5 μm in aerodynamic diameter (PM_2.5_) is a widely used measure of air pollution by agencies such as the United States Environmental Protection Agency (EPA). According to the standards published in 2024, the EPA recommends that PM_2.5_ daily exposure should be below 9 μg/m^3^ for good air quality.^8–10^ Although there have been global data sets from model outputs that provide information on PM_2.5_ distributions, which are key components of pollution monitoring, the lack of validation with ground stations in these data-scarce cities meant that these data sets might not be accurate over Africa or other data-scarce regions.

This study focuses on three of the most populous cities in East Africa: Kampala (Uganda), Nairobi (Kenya), and Dar es Salaam (Tanzania). These cities, like many other in Sub-Saharan Africa, are rapidly expanding and are projected to become some of the largest conurbations in the future. Without targeted policies, the health and environmental burdens of urban air pollution will present a critical challenge there, if not already evident.^11–18^

To address this gap, this study developed a scalable workflow for air quality estimation that does not require extensive technical or computational resources, making it suitable for application in those cities and other data-scarce regions.^19^ By utilizing the proliferation of ground data from low-cost air quality sensors^19–21^ —costing less than USD 2500 apiece—and the extensive spatial coverage of satellite sensors,^22,23^ these data sources were integrated to create localized, ground-validated maps of air quality. These maps enabled the identification of pollution hotspots and seasonal trends, providing insights into the factors driving air pollution in these rapidly growing urban areas. The study further examined spatiotemporal variations in air quality and explored potential causes of the observed patterns.

## Methods and Materials

High-resolution (1 km × 1 km daily) maps of PM_2.5_ from 2019 to 2023 were created and presented as 50 km × 50 km bounding boxes centered over each city. The schematic of the methodology workflow to create those maps is presented in Figure S1 (Supporting Information: methodology workflow). First, processed data from low-cost air quality sensors by AirQo were obtained from 10 stations across Uganda from January 1, 2019 to December 31, 2023 (Supporting Text S1: data sources and Supporting Table S1: data sources). This data has undergone preprocessing with accuracy enhanced by machine learning methods.^24,25^ In addition to this AirQo data set, reference-grade data were acquired; daily PM_2.5_ readings were obtained from one site in Kampala and two sites in Nairobi—all using instruments that had been approved by the United States EPA as Federal Equivalent Methods.

Satellite-based products were obtained off various open-sourced remote sensing products hosted on Google Earth Engine (GEE) (Supporting Text S1: data sources; Supporting Table S2: gridded data sets). Spaceborne measurements of aerosol optical depth (AOD) at 0.47 and 0.55 μm were obtained off the Moderate Resolution Imaging Spectroradiometer (MODIS) platforms.^26^ Data of tropospheric gases—SO_2_, NO_2_, HCHO, CO and O_3_—from the Tropospheric Monitoring Instrument (TROPOMI) on board the Sentinel-5p satellite were also obtained. To investigate land surface changes, monthly values of the Enhanced Vegetation Index (EVI) and Burn Area Index (BAI)—derived also from the MODIS missions—were acquired. These indices are proxies for vegetation coverage and soot signatures, respectively. Also, via the GEE platform, monthly gridded meteorological variables of temperature, relative humidity, winds and precipitation from ERA5-Land^27^ and the Global Precipitation Measurements (GPM) mission^28^ were obtained as auxiliary data sets.

Following preprocessing, the low-cost sensor data were collocated with the daily satellite measurements. After training with a machine-learning approach (Supporting Text S2: creating air quality maps), the resulting algorithm could estimate PM_2.5_ values from the input satellite variables (Supporting Figure S2: observed and estimated PM_2.5_ concentrations). To assess the accuracy of the estimation, the normalized mean biased factor (NMBF) and normalized mean absolute error factor (NMAEF) following the methodology in Yu et al.^29^ were applied (Supporting Text S3: accuracy metrics). Further validation was performed using linear regression against reference monitors in Kampala and Nairobi (reference-grade data were unavailable for Dar es Salaam). After calibration, the estimated PM_2.5_ values have a NMBF of −0.03(0.00) and a NMAEF of 0.29(0.25) when compared to the reference-grade instruments at Kampala (Nairobi) (Supporting Figure S3: observed and estimated PM_2.5_ concentrations). The near-zero NMBF suggests low bias in the estimates, while the NMAEF values indicate an absolute error of approximately 25–29% relative to observed values.

Study boundaries over each city were set at 50 km × 50 km to provide an optimal scale for visualizing the main emission plume, which typically ranged from 20 km to 50 km in diameter.^30^ Then at each 1 km × 1 km pixel, daily PM_2.5_ levels were estimated from satellite-based data retrieved at the same spot. The individual pixels were then mosaiced into a 50 km × 50 km map over each city. At each city, a set of 1825 rasters were generated, representing the daily maps of PM_2.5_ distribution from 2019 to 2023. To analyze the relationship between the land cover indices and meteorological variables, the data sets were collocated to the air quality rasters and correlation coefficients were calculated at the pixel level. Crucially, the estimated values of PM_2.5_ could be lower than actual values as the spatial resolution of the map (1 km × 1 km) was too coarse to identify point pollution hotspots. Another limitation was that the machine-learning algorithm could not consistently reproduce the high concentration levels, because the daily satellite overpass at 1330 h could not capture pollution peaks occurring at other times of the day.

## Results and Discussion

From January 2019 to December 2023, average PM_2.5_ concentrations were 31.4 ± 6.6 μg/m^3^ around Kampala, 21.7 ± 2.8 μg/m^3^ around Nairobi, and 33.1 ± 7.4 μg/m^3^ around Dar es Salaam (Figure 1 and Supporting Figure S4: PM_2.5_ concentrations during 2019–2023). According to the EPA’s 2024 air quality standards, these values fall into the ‘Moderate’ category. Following regional weather patterns, with two dry seasons (the warmer December–February period and the cooler June–August period) and two rainy seasons (March–May and September–November), seasonal variations were observed in the PM_2.5_ concentrations. Higher concentrations occurred during the dry seasons, peaking in July–August and with a smaller peak in January–February. In July–August, average PM_2.5_ concentrations around Kampala and Dar es Salaam were 40.6 and 47.3 μg/m^3^, respectively, categorizing them as ‘Unhealthy for sensitive groups’. In contrast, the air quality around Nairobi was comparatively better, with July–August peak average concentrations at about 27.0 μg/m^3^, within the ‘Moderate’ category.

**Figure 1:**
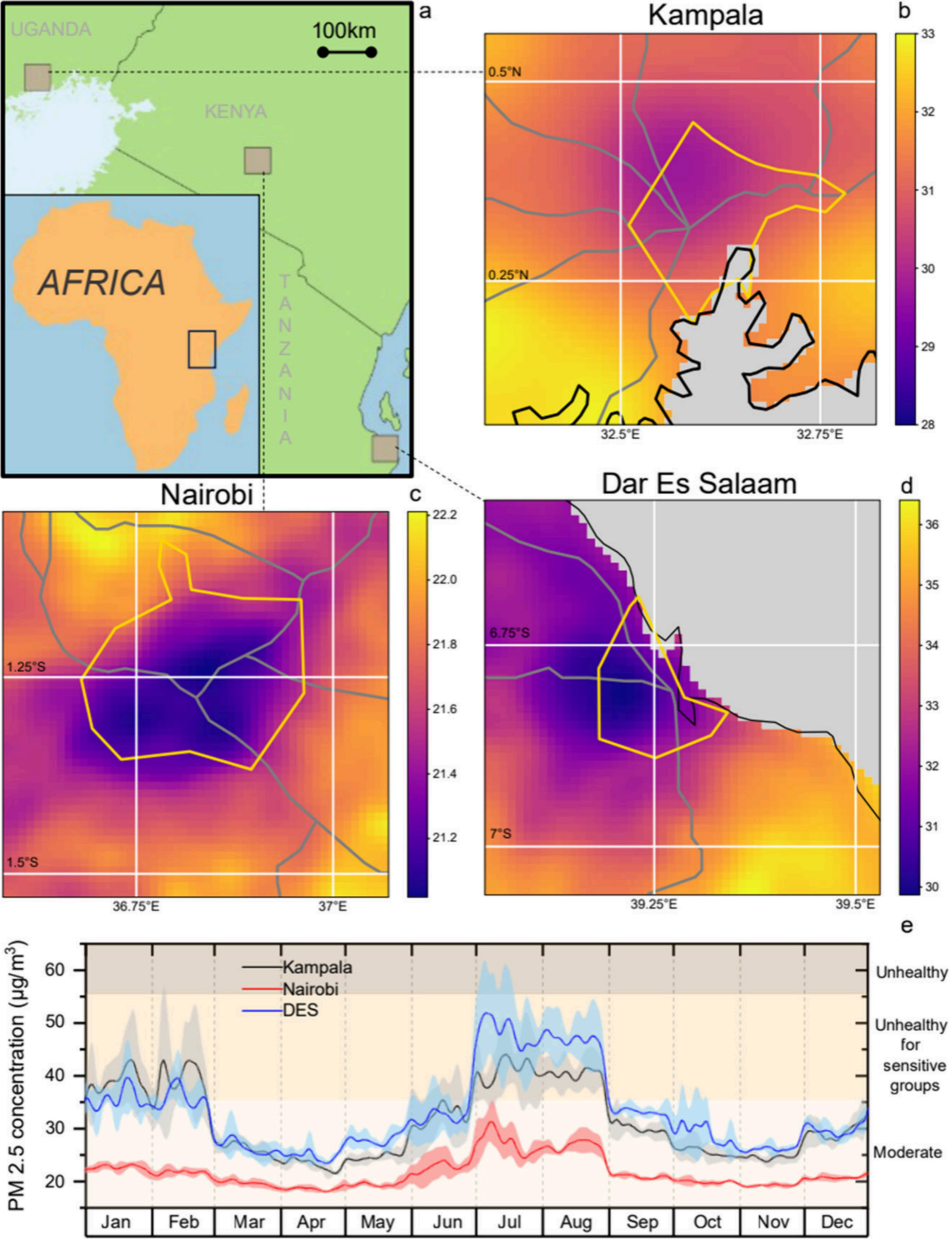
a) Locations of the three urban areas: Kampala, Uganda; Nairobi, Kenya and Dar es Salaam, Tanzania. Estimated PM_2.5_ concentrations (μg/m^3^) aggregated from 2019 to 2023 shown around b) Kampala, c) Nairobi and d) Dar es Salaam (note the different color scales). Gray areas on the map are water bodies, gray lines are major roads and yellow outlines are the urban center boundaries. e) Graph of monthly variation of PM_2.5_ concentrations, averaged over 2019–2023, overlaid atop the air quality standards (Moderate, Unhealthy for sensitive, Unhealthy) set by US’s EPA. Shaded zones indicate ±1 standard deviation.

These seasonal fluctuations in air quality were not uniform spatially across the cities; for example, in July, which had the highest PM_2.5_ levels, marked differences were observed in various sectors (Supporting Figures S5–S7: maps of estimated PM_2.5_ levels). Around Kampala, high concentrations (40–50 μg/m^3^) were seen around Kisubi in the south; around Nairobi, the area adjacent to the C65 highway in the north was a hotspot; around Dar es Salaam, Temeke in the south had PM_2.5_ levels around 60 μg/m^3^, placing air quality there in the ‘Unhealthy’ category. These pollution hotspots also shifted with the seasons. For example, in January, PM_2.5_ concentrations in Waksio in northern Kampala metropolitan area dropped to ∼32 μg/m^3^, while concentrations in the urban center ranged from 40 to 45 μg/m^3^.

Concurring with past studies,^16,17,31^ local meteorology, either directly or through controls on vegetation, was also found to be correlated to PM_2.5_ levels (Supporting Figure S8: meteorological variables and Supporting Table S3: correlation values of meteorological variables with PM_2.5_ concentration). Lower temperatures worsened air quality, especially around Nairobi and Dar es Salaam. Rainfall was inversely correlated with PM_2.5_ levels, likely due to the rainfall scavenging of particulate matter during the rainy season. However, due to the large spatial footprint of the gridded meteorological data sets (∼10 km × 10 km), the impact of urban micrometeorology—such as urban heat islands or street canyoning—could not be captured.

Differences in PM_2.5_ concentrations were also observed between urban centers and suburban zones, with the boundaries defined according to Schneider et al.^32^ (Supporting Text S4: defining urban boundaries). Over the study period of 2019–2023, PM_2.5_ concentrations were significantly lower in urban centers compared to the surrounding suburban zones, as confirmed by a one-tailed *t* test (*p* < 0.05) (Figure 2). During the dry seasons, mean PM_2.5_ concentrations in urban centers were lower by 3.5 μg/m^3^ in Kampala, 2.4 μg/m^3^ in Dar es Salaam, and 0.2 μg/m^3^ in Nairobi. As these values represented averages, the differences were even more pronounced in the pollution hotspots within suburban zones, where concentrations could be 30–80 μg/m^3^ higher. Since the estimations were based on satellite data captured during the midday overpass when much of the city population is concentrated in urban centers, the disparity between urban and suburban zones may be even greater at other times of the day.

**Figure 2:**
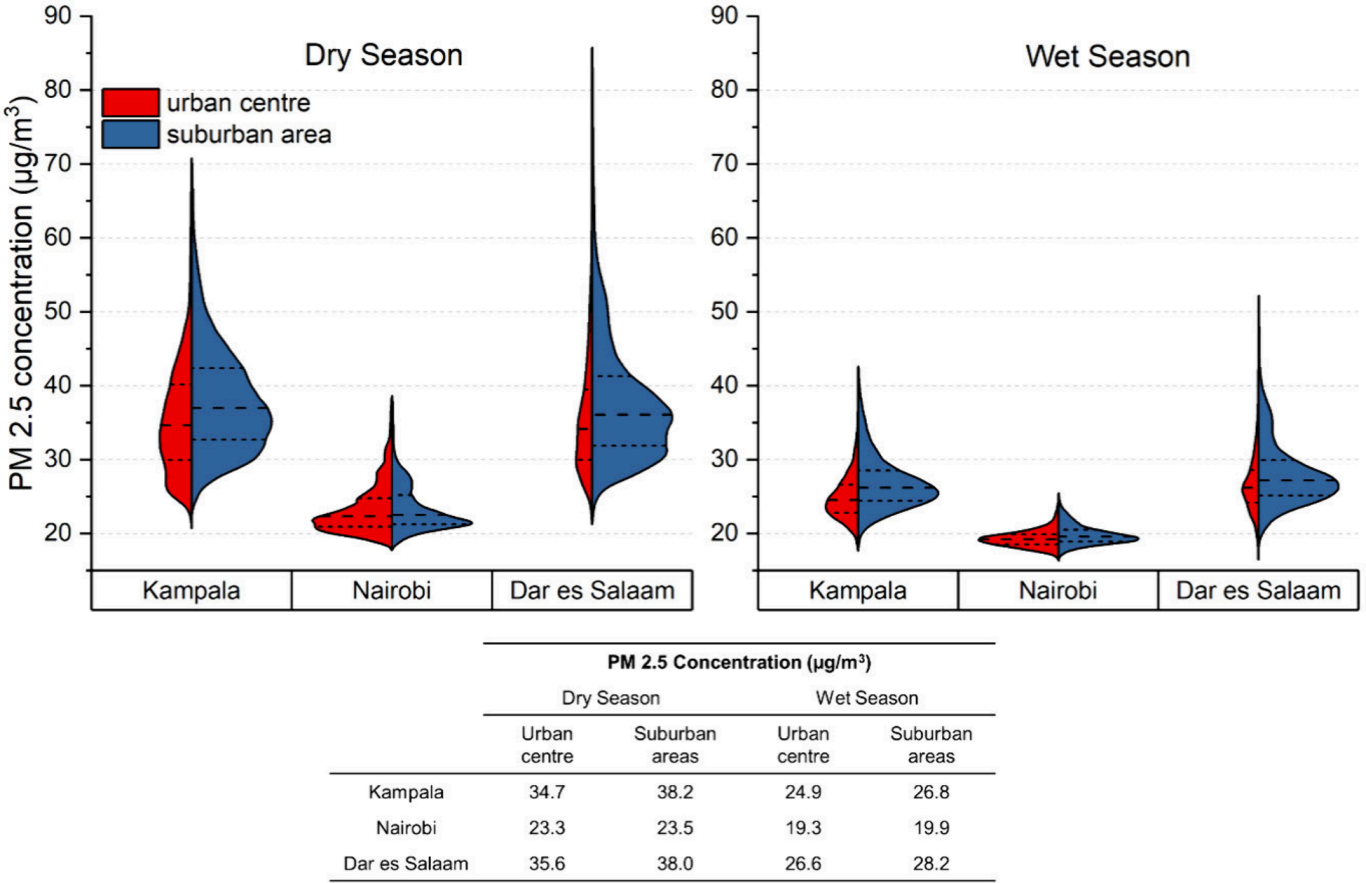
PM_2.5_ concentrations in the urban centers and suburban areas of Kampala, Nairobi and Dar es Salaam during the dry (Dec–Feb, Jun–Aug) and wet seasons (Mar–May, Sep–Nov). Dotted lines on the violin plots indicate the 25th, 50th and 75th percentiles of each respective data set. The table shows the mean PM_2.5_ concentration values from 2019 to 2023 separated by dry–wet seasonality and urban zones.

To investigate further the cause of the poorer air quality in the suburban zones, the air quality maps were compared to the satellite-derived products of Enhanced Vegetation Index (EVI)—a measure of vegetation cover—and the Burn Area Index (BAI)—a proxy of soot signatures. Generally, strong inverse correlations of EVI with PM_2.5_ concentrations were observed especially in the suburban parts of Kampala and Dar es Salaam, suggesting that increasing greenery there was associated with lower PM_2.5_ levels and vice versa (Figure 3). In contrast, the Burn Area Index (BAI)—a satellite-based product that measures soot signatures—showed positive correlations with PM_2.5_ concentrations across all three cities, implying that combustion-related activities were linked to elevated PM_2.5_ levels.

**Figure 3:**
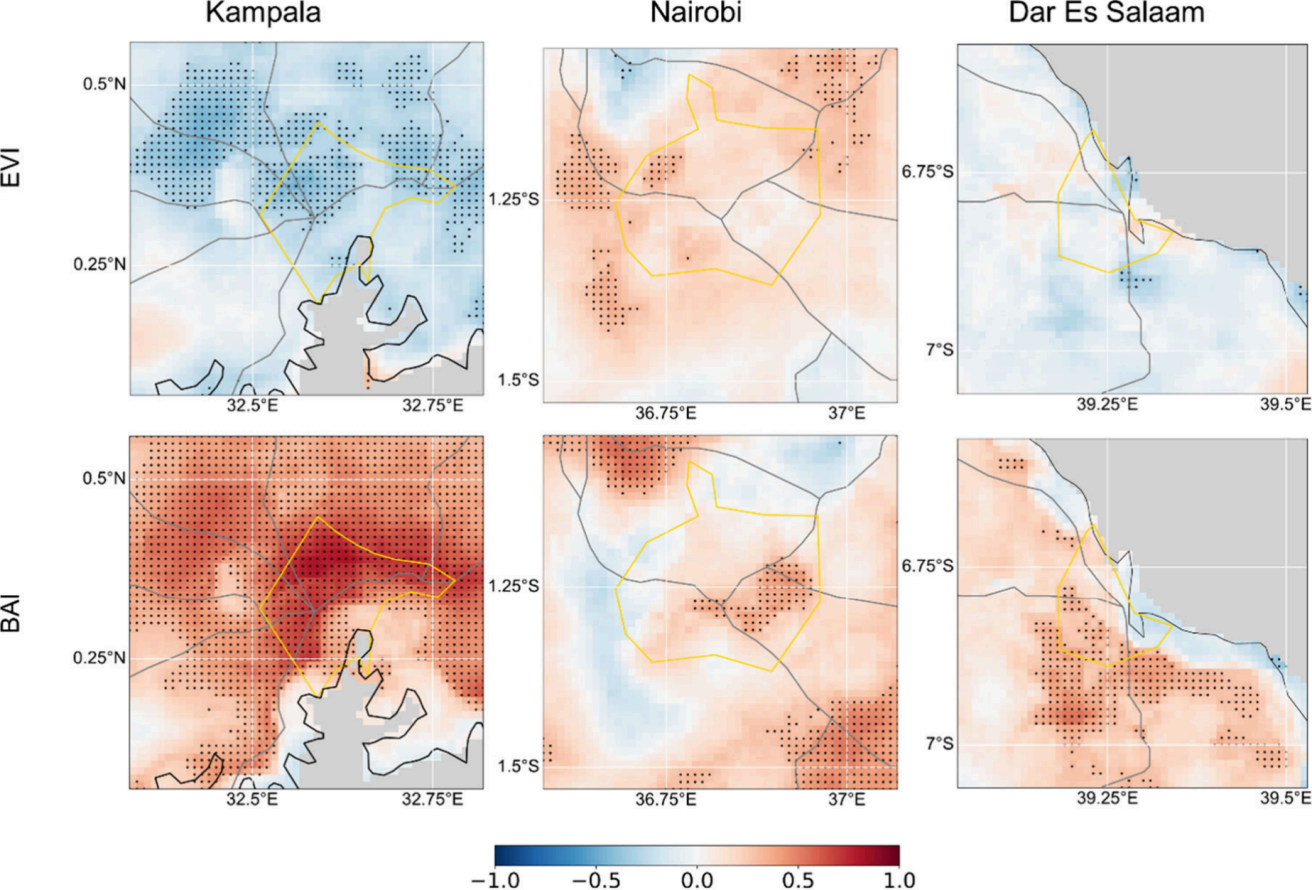
Spearman’s correlation between the PM_2.5_ concentrations and EVI (Enhanced Vegetation Index) and BAI (Burn Area Index). Positive values (red) indicate a direct relationship, while negative values (blue) indicate an inverse relationship. Dots on the figures represent areas where correlations are statistically significant at *p* < 0.05. Gray areas on the map are water bodies, gray lines are major roads and yellow outlines are the urban center boundaries.

Correlation analyses were expanded to the deseasoned residuals of the data sets (Supporting Figure S9: correlation with deseasoned residuals) to distinguish between calendrical patterns and short-term events. A strong correlation with the residual data sets would indicate episodic pollution events, while a strong correlation with the full seasonal data sets would point to underlying seasonal drivers. In Kampala, strong correlations were observed in the seasonal data sets but not in the deseasoned residuals, suggesting a predominantly seasonal relationship between PM_2.5_ and EVI/BAI. In contrast, Dar es Salaam had more significant correlations in the residuals, indicating that short-term, localized events are likely drivers of pollution. Nairobi fell between these two cities, with some neighborhoods showing stronger seasonal correlations and others exhibiting greater correlation with the residuals.

Some areas with strong positive correlations between PM_2.5_ and BAI also displayed strong inverse correlations with EVI, in both the seasonal (Figure 3) and residual data sets (Supporting Figure S9: correlation with deseasoned residuals). Around Nairobi, these areas are clustered in the northern sector around Tigoni, while around Dar es Salaam, in the southern part around Yangeyanye and Temeke. Monthly maps of EVI (Supporting Figures S10–S12: EVI maps) and BAI (Supporting Figures S13–S15: BAI maps) further showed that these suburban areas alternated between stages of greenery in the rainy months and periods with high soot signature in drier months—in contrast to urban centers where less fluctuation were observed. These suburban areas likely became hotspots where dry season conditions intensified the accumulation of soot particles from anthropogenic sources, as the absence of vegetation reduced natural mitigation.^33,34^ Consequently, combustion activities—whether from daily cooking or small-scale land clearing—could lead to substantial soot particle accumulation.^35–38^ Therefore, effective fire management in these suburban areas during the dry season is essential for maintaining cleaner air.

Besides fire-management policies, enhancing urban greenery through the development of parks or the incorporation of vegetation into architectural design can contribute to improved air quality.^12,16,38^ Since other socio-economic factors such as land use change and vehicular traffic have been identified as key drivers of urban air pollution,^15,33,39^ measures such as cleaner fuels or improved traffic management could also potentially improve air quality.^40,41^ Regardless, we reiterate that given the urbanism of African cities, isolated or top-down interventions cannot be panaceas; air quality management must be grounded in integrated, stakeholder-driven approaches to be sustainable and lasting.^19,38,42^

In the future, shifts in commuting patterns in a post-COVID-19 era could result in spatiotemporal migration of air pollution hotspots. Indeed, a recent study found that pandemic-induced mobility restrictions have resulted in lowered PM2.5 concentrations within the Kampala city center,^43^ suggesting that similar effects are occurring in other African cities as well. As our data set straddle the COVID-19 period, there is a likelihood that the urban–suburban air quality disparity observed in this study is already an indicator of changing mobility patterns in a postpandemic era.

By demonstrating a cost-effective approach to integrating and visualizing air quality data, this study offers a framework that can be adapted to other data-scarce regions, such as Southeast Asia or Latin America. Still, these maps—while informative—cannot be taken as absolute reference values for legislation or a complete replacement for ground sensors. Finer spatiotemporal resolution is needed to capture short-term pollution peaks and localized hotspots more accurately. The resolution of our products is currently constrained by the technical limitations of satellite sensors and the resolution and availability of their publicly accessible data. Nonetheless, users interested in just general trends can consider resampling the data to coarser resolutions; for example, aggregating to monthly resolution reduced the NMAEF to 0.13 in Nairobi and 0.17 in Kampala. Conversely, users requiring higher resolution may attempt downscaling the products through integration of various satellite sensors^44^ or calibrating the maps to ground data in real time.^45^ As both upscaling and downscaling involve limitations such as a loss of detail or increased computational demands, these constraints should be carefully considered in relation to specific application needs.

Challenging the assumption that urban centers inherently have worse air quality, we caution that models and studies that claim generic recommendations for urban air quality in Africa may overlook important local processes. Our perspective also suggests that air pollution mitigation efforts, currently focused on urban centers, should be expanded to nearby suburban areas too. Looking ahead, if stakeholders sustain and/or intensify their efforts to improve air quality as these cities continue to expand, there is hope that the African megacities of tomorrow can achieve clean air. The ‘pollution trap’ that afflicts many large cities today could thus be avoided,^13,46^ ultimately creating cities that are “safe, resilient and sustainable” for all.

## Supplementary Material



## Data Availability

Gridded data of MODIS’s aerosol optical depth, Sentinel-5p’s gas concentrations, EVI and BAI indices, and meteorological data set from ERA5-Land and GPM are open access and can be obtained from Google Earth Engine. Code necessary to replicate the results and daily rasters of PM_2.5_ concentrations from 2019 to 2023 over Kampala, Nairobi and Dar es Salaam are available on 10.5281/zenodo.13959948. Please contact the corresponding author for further information if needed.

## Notes

Text S1, data sources; Text S2, creating the air quality maps; Text S3, accuracy metrics; Text S4, defining urban center boundaries and seasonal differences; Figure S1, methodology workflow; Figures S2 and S3, observed and estimated PM_2.5_ concentrations; Figure S4, time series of PM_2.5_ concentrations; Figures S5–S7, estimated PM_2.5_ concentration maps; Figure S8, meteorological variables; Figure S9, correlation with deseasoned residuals; Figures S10–S12, EVI maps; Figures S13–S15, BAI maps; Figure S16, SHAP values of features; Tables S1 and S2, data set used; Table S3, correlation of meteorological variables (PDF)

## References

[1] Dockery D. W., Pope C. A., Xu X., Spengler J. D., Ware J. H., Fay M. E., Ferris B. G., Speizer F. E. (1993). An Association between
Air Pollution and Mortality in Six U.S. Cities.
N Engl J. Med..

[2] Mayer H. (1999). Air Pollution
in Cities. Atmos. Environ..

[3] World Health Organization. WHO Global Air Quality Guidelines: Particulate Matter (PM2.5 and PM10), Ozone, Nitrogen Dioxide, Sulfur Dioxide and Carbon Monoxide; WHO European Centre for Environment and Health: Bonn, Germany, 2021. 34662007

[4] Fisher S., Bellinger D. C., Cropper M. L., Kumar P., Binagwaho A., Koudenoukpo J. B., Park Y., Taghian G., Landrigan P. J. (2021). Air Pollution
and Development in Africa: Impacts on Health, the Economy, and Human
Capital. Lancet Planetary Health.

[5] Rentschler J., Leonova N. (2023). Global Air Pollution Exposure and Poverty. Nat. Commun..

[6] Heft-Neal S., Burney J., Bendavid E., Burke M. (2018). Robust Relationship
between Air Quality and Infant Mortality in Africa. Nature.

[7] Cohen B. (2006). Urbanization
in Developing Countries: Current Trends, Future Projections, and Key
Challenges for Sustainability. Technology in
Society.

[8] Hammer M. S., Van Donkelaar A., Li C., Lyapustin A., Sayer A. M., Hsu N. C., Levy R. C., Garay M. J., Kalashnikova O. V., Kahn R. A., Brauer M., Apte J. S., Henze D. K., Zhang L., Zhang Q., Ford B., Pierce J. R., Martin R. V. (2020). Global Estimates and Long-Term Trends
of Fine Particulate Matter Concentrations (1998–2018). Environ. Sci. Technol..

[9] Mukherjee A., Agrawal M. (2017). World Air Particulate
Matter: Sources, Distribution
and Health Effects. Environ. Chem. Lett..

[10] U.S. Environmental Protection Agency. Technical Assistance Document for the Reporting of Daily Air Quality–the Air Quality Index (AQI); EPA-454/B-24-002; U.S. Environmental Protection Agency: 2024.

[11] Abera A., Friberg J., Isaxon C., Jerrett M., Malmqvist E., Sjöström C., Taj T., Vargas A. M. (2021). Air Quality
in Africa: Public Health Implications. Annu.
Rev. Public Health.

[12] Amegah A. K., Agyei-Mensah S. (2017). Urban Air
Pollution in Sub-Saharan Africa: Time for
Action. Environ. Pollut..

[13] Gani S., Pant P., Sarkar S., Sharma N., Dey S., Guttikunda S. K., AchutaRao K. M., Nygard J., Sagar A. D. (2022). Systematizing
the Approach to Air Quality Measurement and Analysis in Low and Middle
Income Countries. Environ. Res. Lett..

[14] Marais E. A., Silvern R. F., Vodonos A., Dupin E., Bockarie A. S., Mickley L. J., Schwartz J. (2019). Air Quality and Health Impact of
Future Fossil Fuel Use for Electricity Generation and Transport in
Africa. Environ. Sci. Technol..

[15] Dasgupta S., Lall S., Wheeler D. (2021). Spatiotemporal Analysis of Traffic
Congestion, Air Pollution, and Exposure Vulnerability in Tanzania. Science of The Total Environment.

[16] Oguge O., Nyamondo J., Adera N., Okolla L., Okoth B., Anyango S., Afulo A., Kumie A., Samet J., Berhane K. (2024). Fine Particulate Matter
Air Pollution and Health Implications
for Nairobi, Kenya. Environmental Epidemiology.

[17] Okure D., Ssematimba J., Sserunjogi R., Gracia N. L., Soppelsa M. E., Bainomugisha E. (2022). Characterization
of Ambient Air Quality in Selected
Urban Areas in Uganda Using Low-Cost Sensing and Measurement Technologies. Environ. Sci. Technol..

[18] Xu G., Dong T., Cobbinah P. B., Jiao L., Sumari N. S., Chai B., Liu Y. (2019). Urban Expansion
and Form Changes
across African Cities with a Global Outlook: Spatiotemporal Analysis
of Urban Land Densities. Journal of Cleaner
Production.

[19] Bainomugisha E., Adrine Warigo P., Busigu Daka F., Nshimye A., Birungi M., Okure D. (2024). AI-Driven Environmental Sensor Networks and Digital Platforms for
Urban Air Pollution Monitoring and Modelling. Societal Impacts.

[20] Diez S., Lacy S., Coe H., Urquiza J., Priestman M., Flynn M., Marsden N., Martin N. A., Gillott S., Bannan T., Edwards P. M. (2024). Long-Term Evaluation
of Commercial
Air Quality Sensors: An Overview from the QUANT (Quantification of
Utility of Atmospheric Network Technologies) Study. Atmos. Meas. Technol..

[21] Zaidan M. A., Xie Y., Motlagh N. H., Wang B., Nie W., Nurmi P., Tarkoma S., Petaja T., Ding A., Kulmala M. (2022). Dense Air
Quality Sensor Networks: Validation, Analysis, and Benefits. IEEE Sensors J..

[22] Kalisa W., Zhang J., Igbawua T., Henchiri M., Mulinga N., Nibagwire D., Umuhoza M. (2023). Spatial and Temporal Heterogeneity
of Air Pollution in East Africa. Science of
The Total Environment.

[23] Mahmud K., Mitra B., Uddin M. S., Hridoy A.-E. E., Aina Y. A., Abubakar I. R., Rahman S. M., Tan M. L., Rahman M. M. (2023). Temporal
Assessment of Air Quality in Major Cities in Nigeria Using Satellite
Data. Atmospheric Environment: X.

[24] Adong P., Bainomugisha E., Okure D., Sserunjogi R. (2022). Applying Machine
Learning for Large Scale Field Calibration of Low-cost PM _2.5_ and PM _10_ Air Pollution Sensors. Applied AI Letters.

[25] Sserunjogi R., Ssematimba J., Okure D., Ogenrwot D., Adong P., Muyama L., Nsimbe N., Bbaale M., Bainomugisha E. (2022). Seeing the
Air in Detail: Hyperlocal Air Quality Dataset Collected from Spatially
Distributed AirQo Network. Data in Brief.

[26] Lyapustin, A. ; Wang, Y. MODIS/Terra+Aqua Land Aerosol Optical Depth Daily L2G Global 1km SIN Grid V061, 2022. 10.5067/MODIS/MCD19A2.061.

[27] Copernicus Climate Change Service. ERA5-Land Monthly Averaged Data from 1950 to Present, 2019. 10.24381/CDS.68D2BB30.

[28] Huffman, G. J. ; Stocker, E. F. ; Bolvin, D. T. ; Nelkin, E. J. ; Tan, J. GPM IMERG Final Precipitation L3 1 Month 0.1 Degree x 0.1 Degree V07, 2023. 10.5067/GPM/IMERG/3B-MONTH/07.

[29] Yu S., Eder B., Dennis R., Chu S., Schwartz S. E. (2006). New Unbiased
Symmetric Metrics for Evaluation of Air Quality Models. Atmospheric Science Letters.

[30] Lange K., Richter A., Burrows J. P. (2022). Variability
of Nitrogen Oxide Emission
Fluxes and Lifetimes Estimated from Sentinel-5P TROPOMI Observations. Atmos. Chem. Phys..

[31] Mkoma S. L., Kawamura K., Fu P. Q. (2013). Contributions
of Biomass/Biofuel
Burning to Organic Aerosols and Particulate Matter in Tanzania, East
Africa, Based on Analyses of Ionic Species, Organic and Elemental
Carbon, Levoglucosan and Mannosan. Atmos. Chem.
Phys..

[32] Schneider A., Friedl M. A., Potere D. (2009). A New Map of Global
Urban Extent
from MODIS Satellite Data. Environ. Res. Lett..

[33] Meltus Q. J., Karanja F. N. (2024). Mapping Air Quality
Using Remote Sensing Technology:
A Case Study of Nairobi County. OJAP.

[34] Mwangi P. (2024). Evaluating
the Spatio-Temporal Distribution of Nitrogen Dioxide, Land Surface
Temperature and NDVI in Nairobi City County. ISPRS Ann. Photogramm. Remote Sens. Spatial Inf. Sci..

[35] Kirago L., Gatari M. J., Gustafsson Ö., Andersson A. (2022). Black Carbon
Emissions from Traffic Contribute Substantially to Air Pollution in
Nairobi, Kenya. Commun. Earth Environ.

[36] Nix E., Nabukwangwa W., Mwitari J., Lorenzetti F., Gohole A., Saligari S., Shupler M., Abbott M., Rosa G., Anderson
De Cuevas R., Nyongesa M., Puzzolo E., Pope D. (2024). ‘This
Smoke Will Finish Us’: Impacts of Cooking with
Polluting Fuels on Air Quality, Health and Education in Three Schools
in Nairobi, Kenya. Environ. Res.: Health.

[37] Okello G., Nantanda R., Tatah L., Sserunjogi R., Johnson O., Awokola B., Okure D., Thondoo M., Green P., Babajide O., Oni T. (2024). Association
between
Ambient Air Pollution and Respiratory Health in Kampala, Uganda: Implications
for Policy and Practice. Urban Climate.

[38] Okure D., Guttikunda S. K., Sserunjogi R., Adong P., Dammalapati S. K., Lsoto D., Green P., Bainomugisha E., Xie J. (2025). Integrated Air Quality Information for Kampala: Analysis of PM_2.5_, Emission Sources, Modelled Contributions, and Institutional
Framework. Environ. Sci.: Atmos..

[39] Matara C., Osano S., Yusuf A., Akech E. (2024). An Assessment of the
Contribution of Vehicular Traffic to Ambient Air Quality - A Case
Study of Nairobi Expressway Corridor. Civil
and Environmental Engineering.

[40] Rajé F., Tight M., Pope F. D. (2018). Traffic
Pollution: A Search for Solutions
for a City like Nairobi. Cities.

[41] Wang L., Chen X., Zhang Y., Li M., Li P., Jiang L., Xia Y., Li Z., Li J., Wang L., Hou T., Liu W., Rosenfeld D., Zhu T., Zhang Y., Chen J., Wang S., Huang Y., Seinfeld J. H., Yu S. (2021). Switching to Electric Vehicles Can
Lead to Significant Reductions of PM2.5 and NO2 across China. One Earth.

[42] Garland R. M., Altieri K. E., Dawidowski L., Gallardo L., Mbandi A., Rojas N. Y., Touré N. E. (2024). Opinion:
Strengthening Research in
the Global South–Atmospheric Science Opportunities in South
America and Africa. Atmos. Chem. Phys..

[43] Ghaffarpasand O., Okure D., Green P., Sayyahi S., Adong P., Sserunjogi R., Bainomugisha E., Pope F. D. (2024). The Impact of Urban
Mobility on Air Pollution in Kampala, an Exemplar Sub-Saharan African
City. Atmospheric Pollution Research.

[44] Zou B., Liu N., Li Y., Zang Z., Li S., Li S., Wu J. (2024). Generating
Hourly Fine Seamless Aerosol Optical Depth Products by
Fusing Multiple Satellite and Numerical Model Data. IEEE Trans. Geosci. Remote Sensing.

[45] Schulte N., Li X., Ghosh J. K., Fine P. M., Epstein S. A. (2020). Responsive High-Resolution
Air Quality Index Mapping Using Model, Regulatory Monitor, and Sensor
Data in Real-Time. Environ. Res. Lett..

[46] Kulmala M., Kokkonen T. V., Pekkanen J., Paatero S., Petäjä T., Kerminen V.-M., Ding A. (2021). Opinion: Gigacity–a Source
of Problems or the New Way to Sustainable Development. Atmos. Chem. Phys..

