# Supplementary material for: East African City Centers Show Lower PM2.5 Levels than Their Suburbs
Source: Environ Sci Technol Lett. 2025 Jun 9;12(9):1169–76. doi: 10.1021/acs.estlett.5c00451 (PMC12424180; doi:10.1021/acs.estlett.5c00451)
Supplement: Supplementary file 1 [file ez5c00451_si_001.pdf]

Supplementary Information for

East African city centres show lower $PM_{2.5}$ levels than their suburbs

Samuel De Xun Chua[1,2*], Otienoh Oguge[3], Celestine Atieno Oliewo[4], Richard Sserunjogi[5], Deo Okure[5], Priscilla Adong[5], Asinta Manyele[6], Tareq Hussein[1], Yuheng Yang[2], Xixi Lu[2], Katrianne Lehtipalo[1,7], Martha Arbayani Zaidan[1,8], Tuukka Petäjä[1]

*Corresponding author: samuel.chua@helsinki.fi

[1]Institute for Atmospheric and Earth System Research, Faculty of Science, University of Helsinki, Helsinki, 00014, Finland.
[2]Department of Geography, National University of Singapore, Singapore, 119077, Singapore.
[3]Eastern Africa GEOHealth Hub, Centre for Advanced Studies in Environmental Law and Policy, Faculty of Law, University of Nairobi, Nairobi, 00100, Kenya
[4]"Enzo Ferrari" Department of Engineering, University of Modena and Reggio Emilia, Modena, 41121, Italy.
[5]AirQo, Department of Computer Science, Makerere University, Kampala, 7062, Uganda
[6]Electronics and Telecommunication Engineering, Dar es Salaam Institute of Technology, Dar es Salaam, 2958, Tanzania
[7]Finnish Meteorological Institute, Helsinki, 00101, Finland
[8]Department of Computer Science, Faculty of Science, University of Helsinki, Helsinki, 00014, Finland.

Contents of this file

Text S1 to S4

Figure S1 to S16

Table S1 to S3

Supporting References

Text S1: Data sources

Ground-based air quality measurements were obtained from various locations across eastern Africa (Table S1). Within eastern Africa, the network of low-cost sensors operated by AirQo[1] is one of the most widespread in spatiotemporal coverage. These sensors incorporated dual Plantower Sensors (PMS 5003) and a laser scattering technique to derive $PM_{2.5}$ concentrations[2]. Preprocessed data, prepared with the protocol described in Adong et al.[3], were obtained from the AirQo portal.

For the reference-grade data, daily $PM_{2.5}$ data were collected from a reference site in Kampala that operated the Met One Beta Attenuation Monitor Model 1022 (BAM-1022). Similarly, daily $PM_{2.5}$ readings were obtained from two sites in Nairobi. At the University of Nairobi site, a BAM-1022 was used, while at the US Embassy in Nairobi, a Teledyne T640 PM Mass Monitor was employed. Both instruments were approved by the United States Environmental Protection Agency as Federal Equivalent Methods, thus ensuring their reliability [4].

Various gridded datasets were obtained from the open-sourced collections available in Google Earth Engine (Table S2). Measurements of aerosol optical depth at 0.47µm and 0.55µm that were obtained off the MODIS platforms had been processed with the Multi-angle Implementation of Atmospheric Correction (MAIAC) C6.1 algorithm [5], which has demonstrated comparable or better performance than other commonly used algorithm such as Deep Blue or Dark Target [6,7]. Concurrently, tropospheric gas data from the Sentinel-5p mission, namely: SO_2, NO_2, HCHO, CO and O_3 concentrations, were obtained. Both satellites have revisit times of approximately once a day at around 1330 hours.

The Enhanced Vegetation Index (EVI) and Burn Area Index (BAI) that was obtained off the MODIS platform had been calculated following established methods [8,9]. Normal standardisation in accordance with Eq. S1 was performed for raw values of BAI at each city, to constrain most values of BAI to ±1 for more intuitive visualisation.

$$Z = \frac{x - \mu}{\sigma}$$	Eq. S1

where Z is the standardised output, x is the raw value µ is the mean value and σ is the standard deviation. The normalised BAI outputs had a range of roughly -1 to 1, corresponding to ±1 standard deviation around the mean.

Measuring burned areas in satellite imagery has been a challenge with various indices being proposed. Among them, the BAI were chosen for its ease in calculation and its relatively good performance in discriminating the soot spectral signature [9,10]. However, a major limitation is that the BAI has problem identifying water bodies from burned areas. Therefore, for our study, the areas adjacent to water of Kampala and Dar es Salaam showed high values of BAI. There, the high values were likely due to the capture of pixels with high water spectral signature. Nonetheless, the BAI is still a good measure of burned areas in areas inland without big water bodies. Despite this limitation, the results of the study remained valid since the areas that showed high correlation of BAI with $PM_{2.5}$ concentrations were located inland, away from contamination by water pixels. However, there is a possibility that the area of burned land is underestimated, as combustion activities may occur in the coastal regions.

Monthly values of temperature, dewpoint temperature, and the x and y components of surface wind was acquired from the ERA5-Land reanalysis product at 11km x 11km resolution [11]. For rainfall data, monthly values at about 11km x 11km resolution were downloaded from data processed by the Integrated Multi-satellite Retrievals for GPM (IMERG) algorithm, that is part of the Global Precipitation Measurement (GPM) mission [12]. Subsequently, relative humidity (RH) values were calculated off the temperature(T) and dewpoint temperature (T_d) values as per Eq. S2:

$$RH = 100exp^{\left(\frac{\lambda\beta(T_d - T)}{(\lambda+T)(\lambda+T_d)}\right)}$$	Eq. S2

where β = 17.625 and λ = 243.04°C following empirical tests [13]. Wind speed and wind direction were calculated respectively off the magnitudes and angle of the x and y components of surface wind.

Text S2: Creating the air quality maps

Although the data from the low-costs sensors have been calibrated, their high temporal frequencies of subhourly measurements and long deployment times meant that stochastic errors are inevitable [14,15]. Two criteria were used to identify outlier data: 1. Data should not be too extreme and 2. The derivative with respect to time should not be too sharp. In other words, data experiencing overly large jumps/drops within a short time period are probably erroneous. To eliminate those outliers, we employed an unsupervised machine learning method, DBSCAN, to identify anomalous datapoints based on the two criteria. DBSCAN is a density-based clustering algorithm with strong ability to differentiate noise from data [16], rendering it a suitable method in our situation.

After removing the outlier data from the low-cost sensors, an algorithm was needed to correlate the satellite-based variables to the ground-based air quality data. To create the training datasets, the ground-based air quality measurements were first aggregated into daily levels. The ground sensors were then collocated with satellite measurements for that day. While the spaceborne sensors might be able to 'see' through light cloud cover, they were still affected by thick surface clouds that are common in the study areas, causing data gaps. Thus, several methods were used to fill up some of the gaps. For example, in the scenario that a 1x1 pixel did not have a measurement, the mean values of the neighbouring 3x3 pixels would be used to fill up the gap instead. Temporal gaps at a single pixel were interpolated for a maximum of four days using spline fitting across the entire timeseries (2019-2023) at that specific pixel. The resulting dataset of 11995 entries was then split into training and testing sets in the ratio of 80:20.

As various studies had shown the seasonality of air quality in eastern African cities [2,17,18], month was incorporated as an independent variable. To handle this combination of categorical (month) and continuous variables (the satellite variables of AOD at both 0.47µm and 0.55µm, and tropospheric gas concentrations – SO_2, NO_2, HCHO, CO and O_3) as independent variables within a regression algorithm, a machine-learning approach was chosen. The training dataset were trained using the eXtreme Gradient Boosting (XGBoost) algorithm. XGBoost offers an advantage over conventional statistical models due to its capability to identify trends through the construction of an ensemble of pseudo-decision trees. This approach enabled XGBoost to achieve reasonable accuracy without requiring excessive computational resources [19]. Furthermore, XGBoost could handle data gaps by ignoring missing satellite input values. Optimisation was performed with 10-fold cross validations.

Estimated values from the satellite-air quality algorithm were subsequently compared to ground reference data and further calibrated using linear regression curves. In greater detail, actual values were plotted against estimated values. To the scatterplot, a linear regression curve was fitted. The equations of the linear line, expressing actual values (y) as a function of the estimated values (x), were thus also the calibration equations (Eq. S3- S4). Since reference monitors were unavailable for Dar es Salaam, calibration was not possible there.

$y_{kampala} = 2.59 + 0.8179x_{kampala}$	Eq. S3
$y_{nairobi} = 10.86 + 0.341x_{nairobi}$	Eq. S4

To estimate the feature importance of the various variables in the $PM_{2.5}$ estimation algorithm, the Shapley Additive Explanations (SHAP) framework was applied [20]. SHAP values serve as proxies for contributions of the independent variable to the estimated results, enhancing the explainability of the machine learning algorithm—in this case, XGBoost. In order of decreasing importance, the most influential features were CO number density, NO_2 number density, SO_2 number density, HCHO number density, O_3 number density, month, Optical Depth at 0.47 µm, and Optical Depth at 0.55 µm (Figure S16). However, note that the XGBoost algorithm does not account for collinearity among input variables. As a result, when variables are correlated (e.g., Optical Depth at 0.47 µm and Optical Depth at 0.55 µm), the importance of one feature may be overestimated at the expense of the other.

Text S3: Accuracy metrics

To assess the accuracy of the estimation, the normalized mean biased factor (NMBF) and normalized mean absolute error factor (NMAEF) following the methodology in Yu et al. [21] were applied. These accuracy metrics were used to better capture the scale and distribution of the errors from air quality sensors [21–23]. NMBF was calculated as per Eq. S5 while NMAEF was calculated as per Eq. S6.

$NMBF = \frac{\sum(E-O)}{\sum O} \text{ if } \overline{E} \geq \overline{O}$ $NMBF = \frac{\sum(E-O)}{\sum E} \text{ if } \overline{E} < \overline{O}$	Eq. S5
$NMAEF = \frac{\sum\lvert E-O\rvert}{\sum O} \text{ if } \overline{E} \geq \overline{O}$ $NMAEF = \frac{\sum\lvert E-O\rvert}{\sum E} \text{ if } \overline{E} < \overline{O}$	Eq. S6

E and O refer to estimated and observed values respectively.

NMBF ranges from -∞ to +∞ and can be regarded as the bias of estimation with respect to observations. When NMBF = 0, it means that there is no bias, while positive and negative values imply overestimation and underestimation respectively. NMAEF ranges from 0 to +∞ with values implying the magnitude of the factor by which estimations err from observations. For instance, a value of 0.5 would imply that the mean error is about 1.5 times the mean observation.

Text S4: Defining urban centre boundaries and seasonal differences

Across the study areas in Kampala, Nairobi, and Dar es Salaam, a clear visual distinction between the denser urban centres and the more open suburban zones could be observed. However, these urban characteristics might not align with official administrative boundaries set by city governments, making it challenging to objectively demarcate the two urban zones using official borders.

To address this, the widely accepted boundaries defined by Schneider et al.[24] were used to distinguish between the urban centres and their surrounding suburban zones. Those boundaries had been created by combining MODIS-based images with inputs of expected city and vegetation structure to generate maps of urban areas where anthropogenic surfaces dominated[24]. Although the dataset was generated in 2009, it remained relevant for this study. The boundaries defined by Schneider et al. still represent the denser and more established urban cores, which contrast with the more open surrounding suburban zones that may have developed more recently. These boundaries also corresponded well with present-day ground conditions — the city proper is located within the urban centre boundaries, while the surrounding metropolitan area and suburbs fall outside those limits.

Following the urban boundaries, $PM_{2.5}$ data were categorised into urban and suburban zones as well as dry and wet seasons. A one-tailed t-test with Welch's correction was applied at 0.05 significance level to evaluate whether mean $PM_{2.5}$ values in suburban areas were significantly higher than those in urban areas during the respective seasons. In other words, the test assessed whether $PM_{2.5}$ levels in suburban zones exceeded those in urban centres. Welch's correction was applied because equal variance was not assumed, although the analyses was performed on entire populations rather than samples. The data distribution and key percentiles are illustrated in Figure 2. The statistical analyses were performed using *OriginPro 2024b*.

The meteorological conditions in the study can be categorized into two dry seasons (a warmer period from December to February and a cooler period from June to August) and two rainy seasons (March to May and September to November). One-tailed t-tests with Welch's correction were applied at a 0.05 significance level to determine whether mean $PM_{2.5}$ levels during the dry seasons were significantly higher than those during the wet seasons. Significant differences ($p<0.05$) in $PM_{2.5}$ levels were observed across all three cities.

De-seasoning of the $PM_{2.5}$ concentration, EVI, and BAI datasets was also performed to determine whether the observed correlations were driven by seasonal patterns. Residual datasets were generated using seasonal trend decomposition with LOWESS (locally weighted scatterplot smoothing) from the Python module *statsmodels v0.15.0*. This algorithm removes the underlying trend and seasonal components from each pixel's time series, leaving only the de-seasoned residuals.

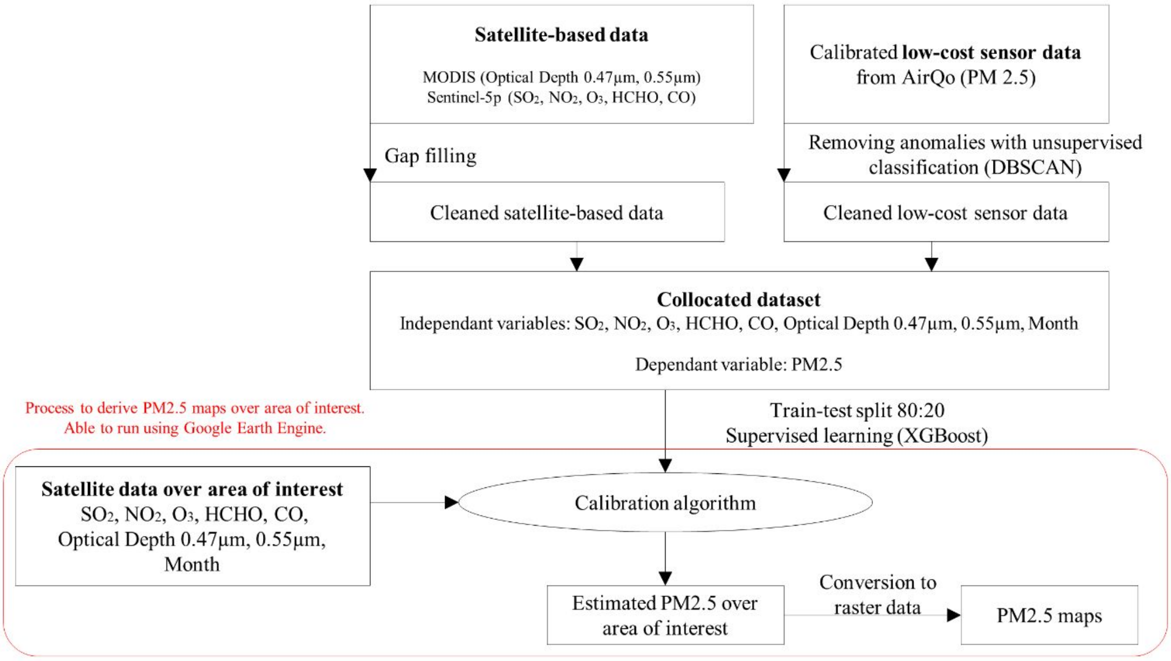

Figure S1. Methodology workflow in this study. Red box denotes process that generates $PM_{2.5}$ maps from satellite data that could be run on Google Earth Engine cloud. Code needed for this process is available in the *Data and code availability* section.

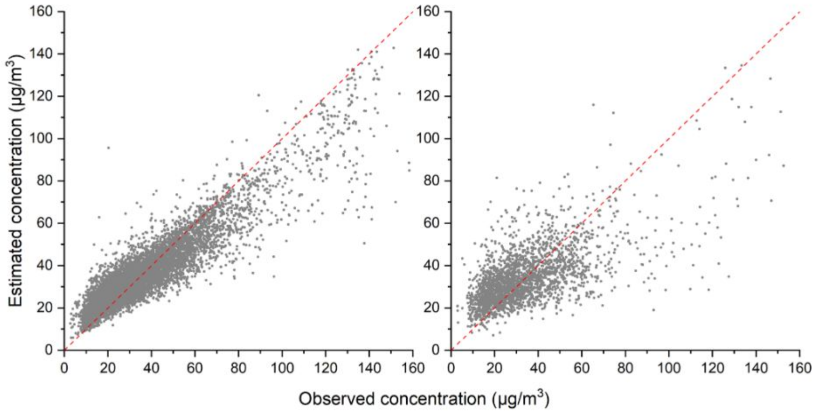

	Training	Testing
R^2	0.82	0.42
NMBF	0.00	0.00
NMAEF	0.18	0.32

Figure S2. Observed and estimated concentrations of $PM_{2.5}$ concentrations on the training and testing dataset. Accuracy metrics of r-squared values (R^2), normalised mean bias factor (NMBF) and normalised mean absolute error factor (NMAEF) are also shown. Refer to Text S3 on how NMBF and NMAEF are calculated.

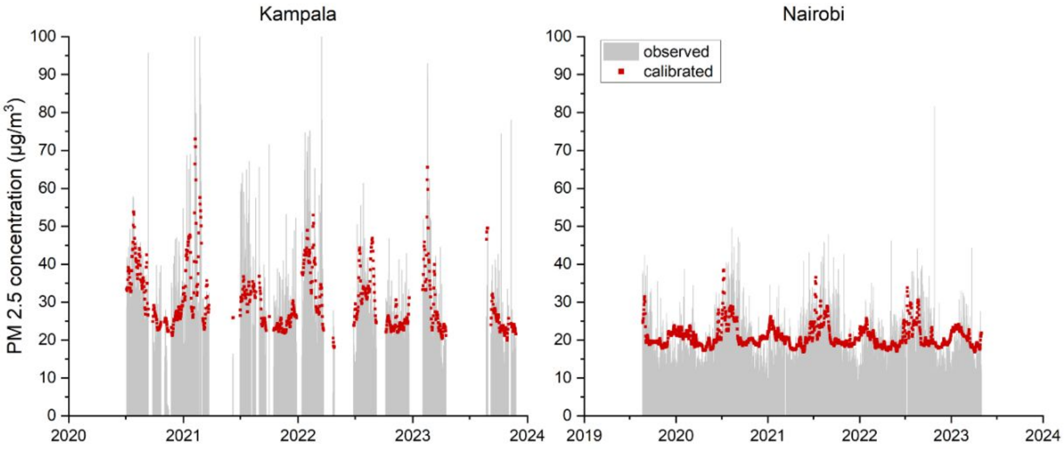

	Kampala	Nairobi
NMBF	0.03	0.00
NMAEF	0.29	0.25

Figure S3. Time series of estimated $PM_{2.5}$ concentrations (red dots) after further calibration with ground reference-level monitors (grey bars). Accuracy metrics of normalised mean bias factor (NMBF) and normalised mean absolute error factor (NMAEF) are also shown. Refer to Text S3 on how NMBF and NMAEF are calculated.

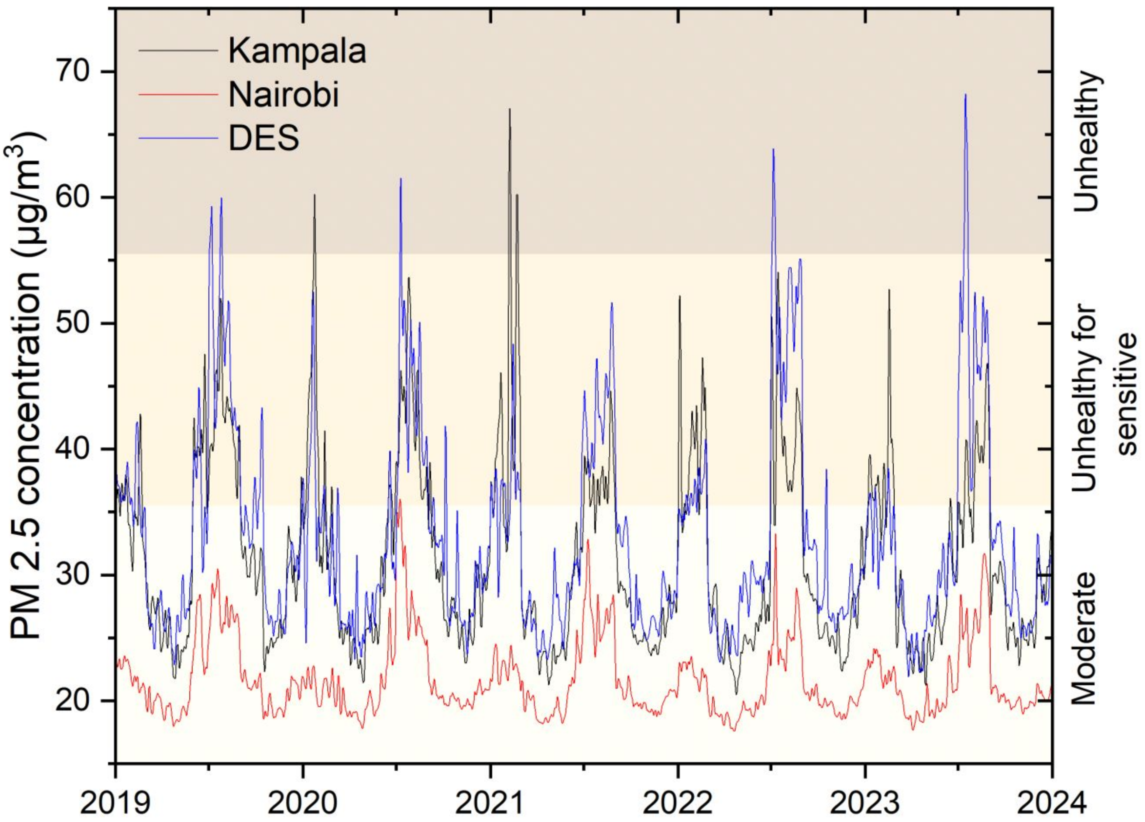

Figure S4. Timeseries of $PM_{2.5}$ concentrations during 2019-2023 estimated at Kampala, Nairobi and Dar es Salaam. According to the US EPA's guidelines for air quality, the boundaries for 'Moderate', 'Unhealthy for sensitive groups' and 'Unhealthy' are shown in the background.

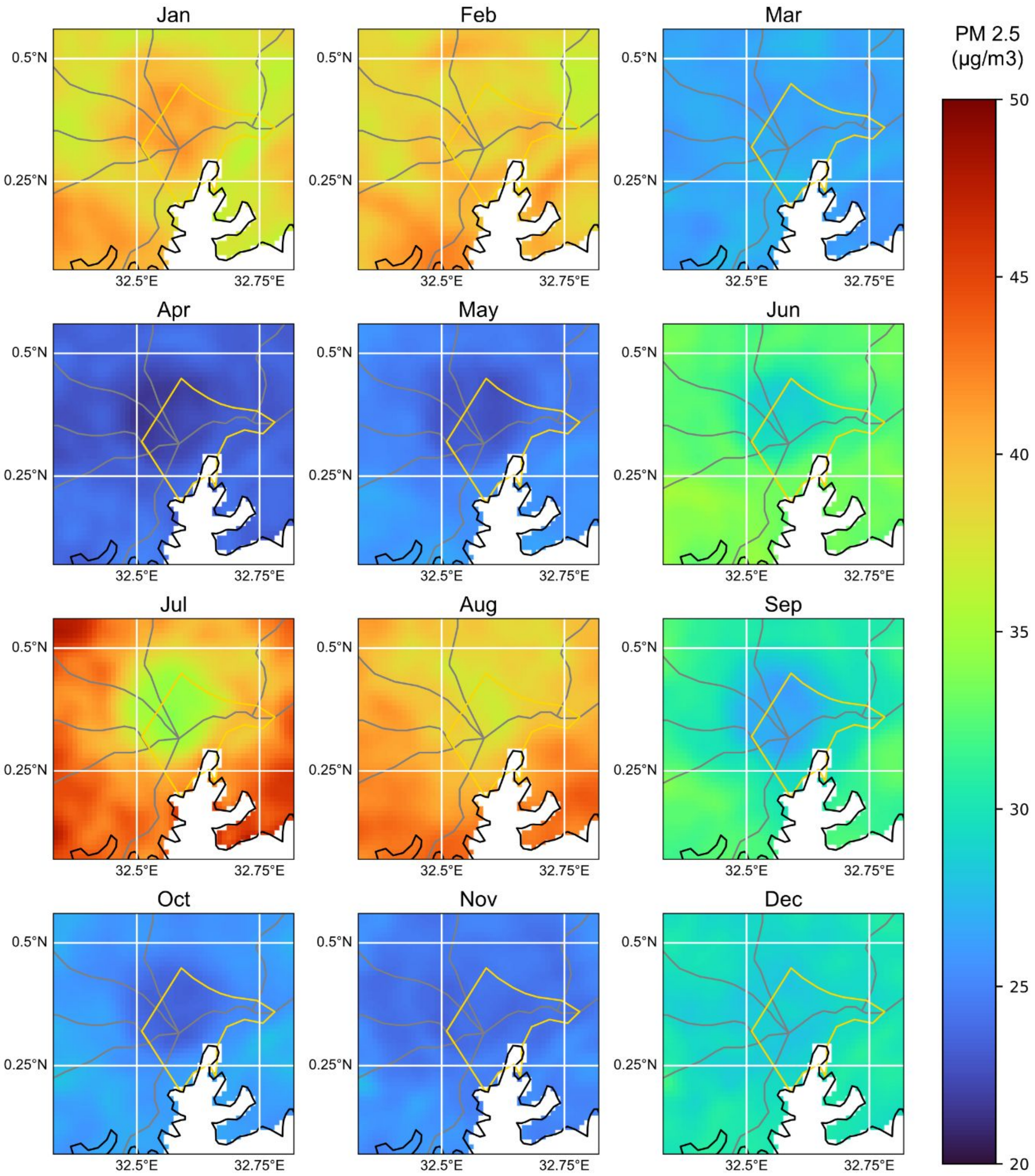

Figure S5. Estimated $PM_{2.5}$ levels during 2019-2023 aggregated by months at Kampala. White areas on the map indicate water areas, grey lines indicate major roads and the yellow outline indicate the urban centre boundary.

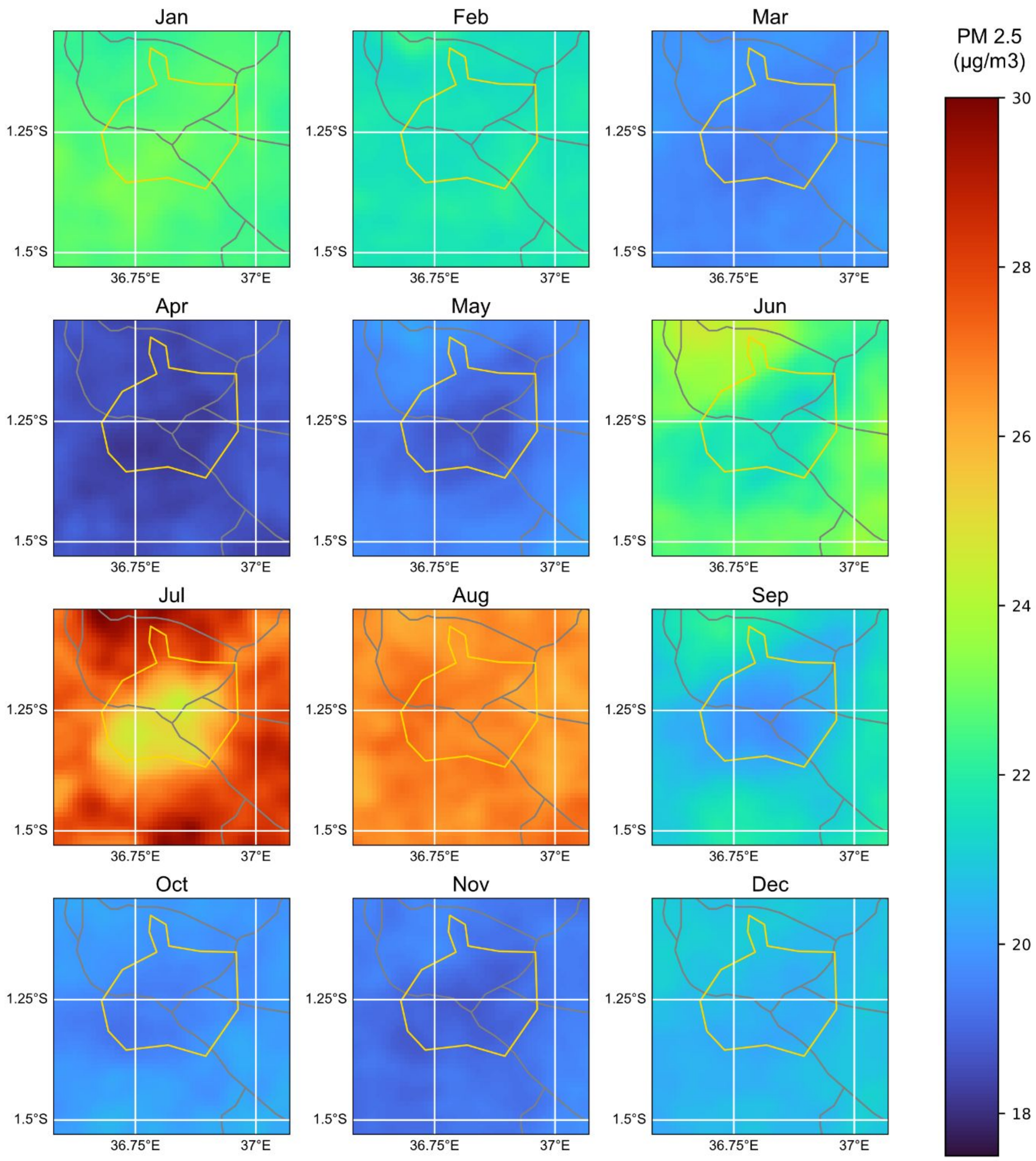

Figure S6. Estimated $PM_{2.5}$ levels during 2019-2023 aggregated by months at Nairobi. Grey lines indicate major roads and the yellow outline indicate the urban centre boundary.

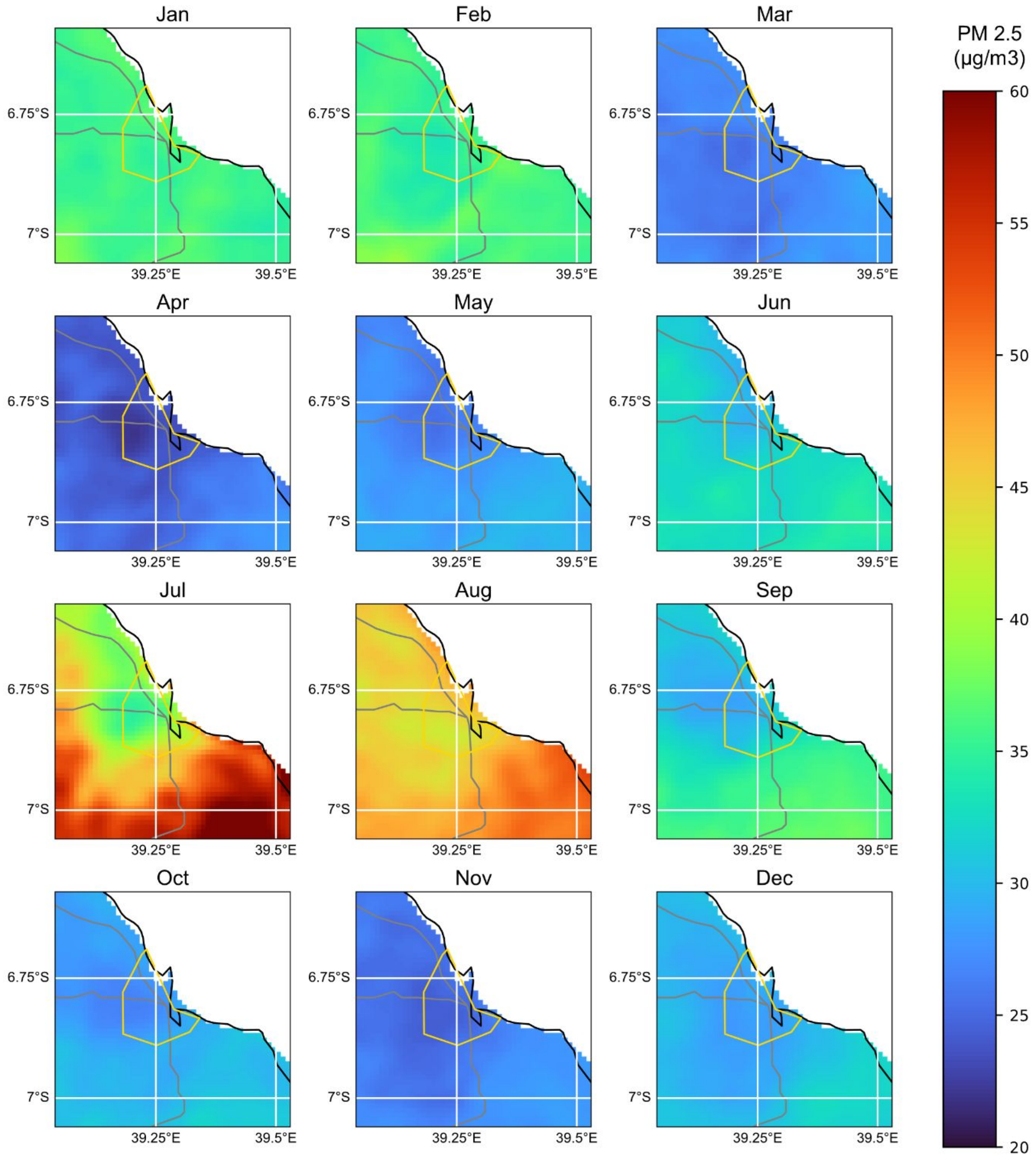

Figure S7. Estimated $PM_{2.5}$ levels during 2019-2023 aggregated by months at Dar es Salaam. White areas on the map indicate water areas, grey lines indicate major roads and the yellow outline indicate the urban centre boundary.

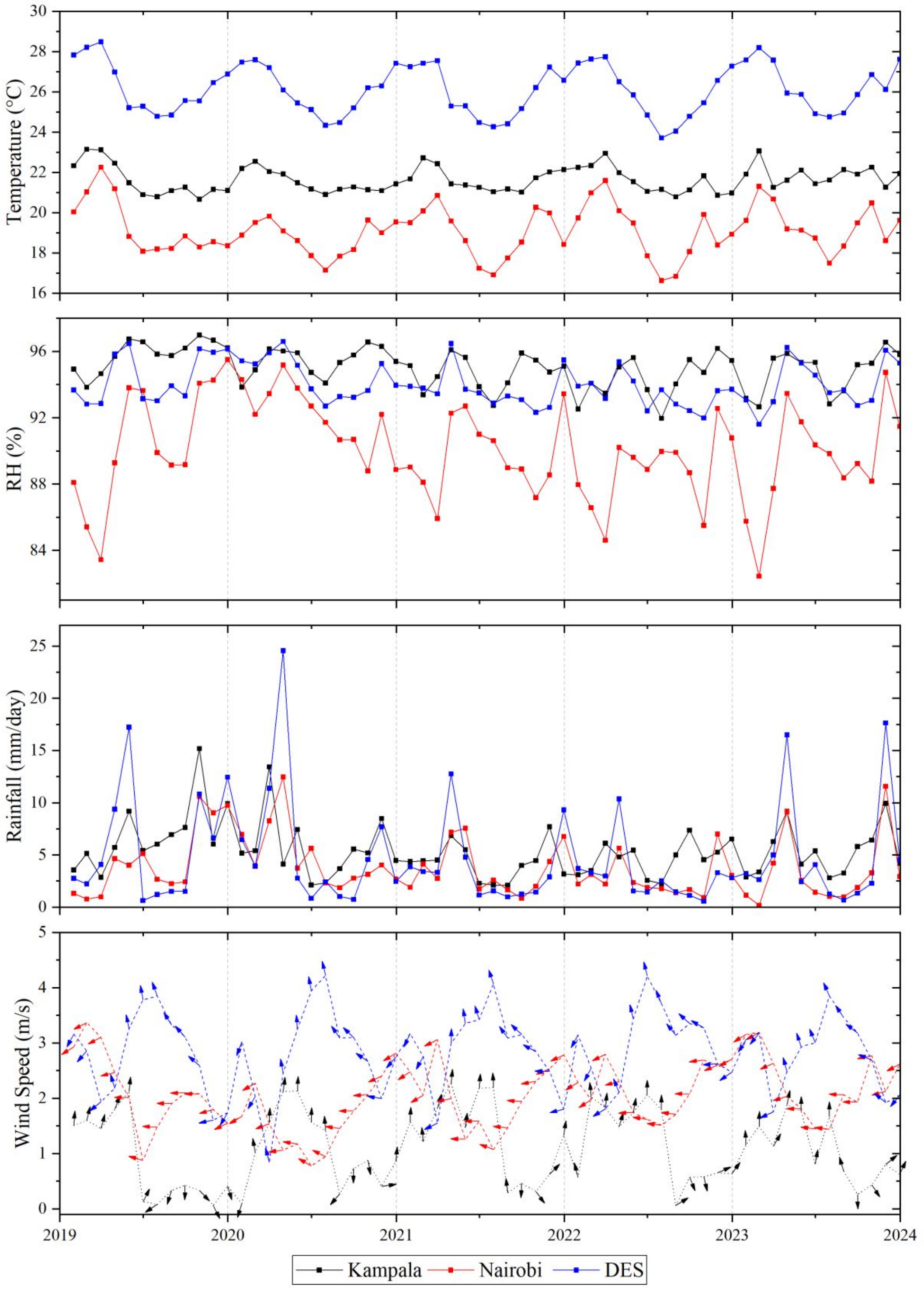

Figure S8. Time series of meteorological variables at Kampala, Nairobi and Dar es Salaam from 2019 to 2024. From top to bottom, the variables are air temperature, relative humidity, rainfall and windspeed. For the wind speed plot, prevailing wind direction is shown as arrowhead directions.

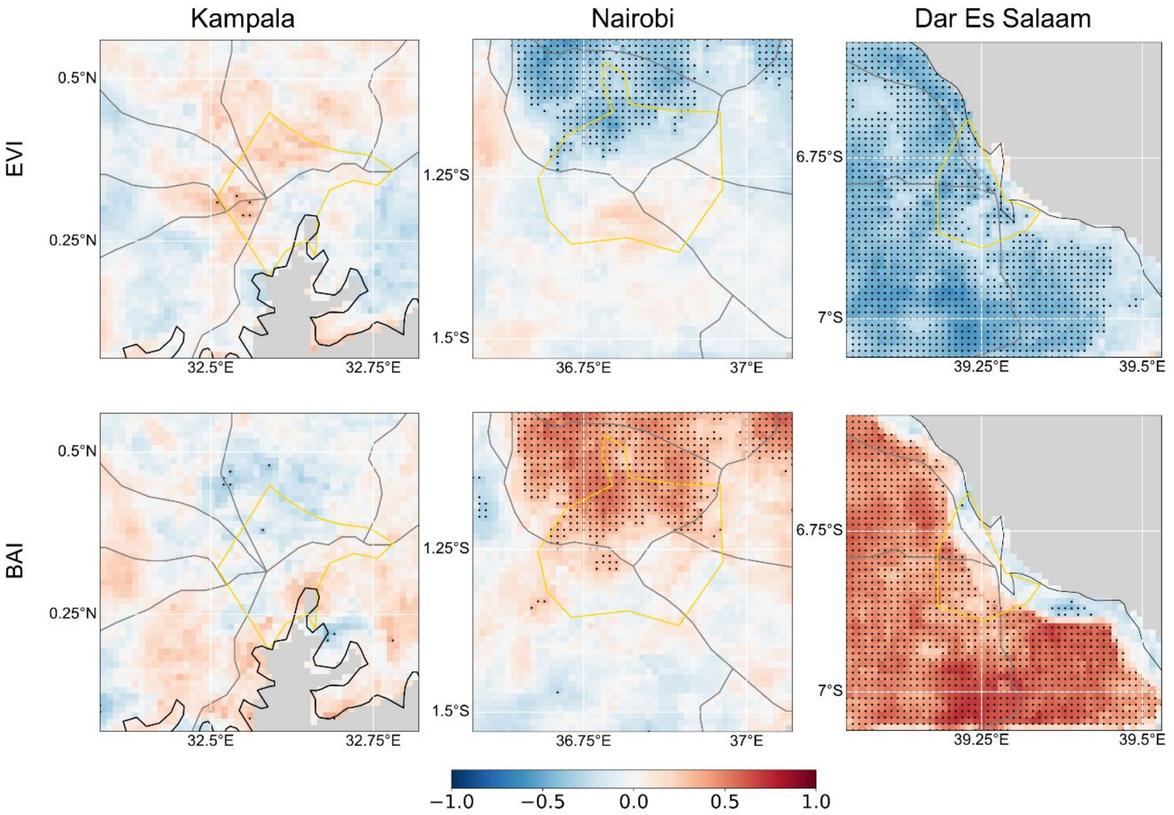

Figure S9. Spearman's correlation between the de-seasoned residuals (Text S4) of $PM_{2.5}$ concentrations, and EVI (Enhanced Vegetation Index) and BAI (Burn Area Index). Positive values (red) indicate direct relationship while negative values (blue) indicate inverse relationship. Dots on the figures represent areas where correlation are statistically significant at $p<0.05$. Grey areas on the map are water bodies; grey lines are major roads and yellow outlines are the urban centre boundaries.

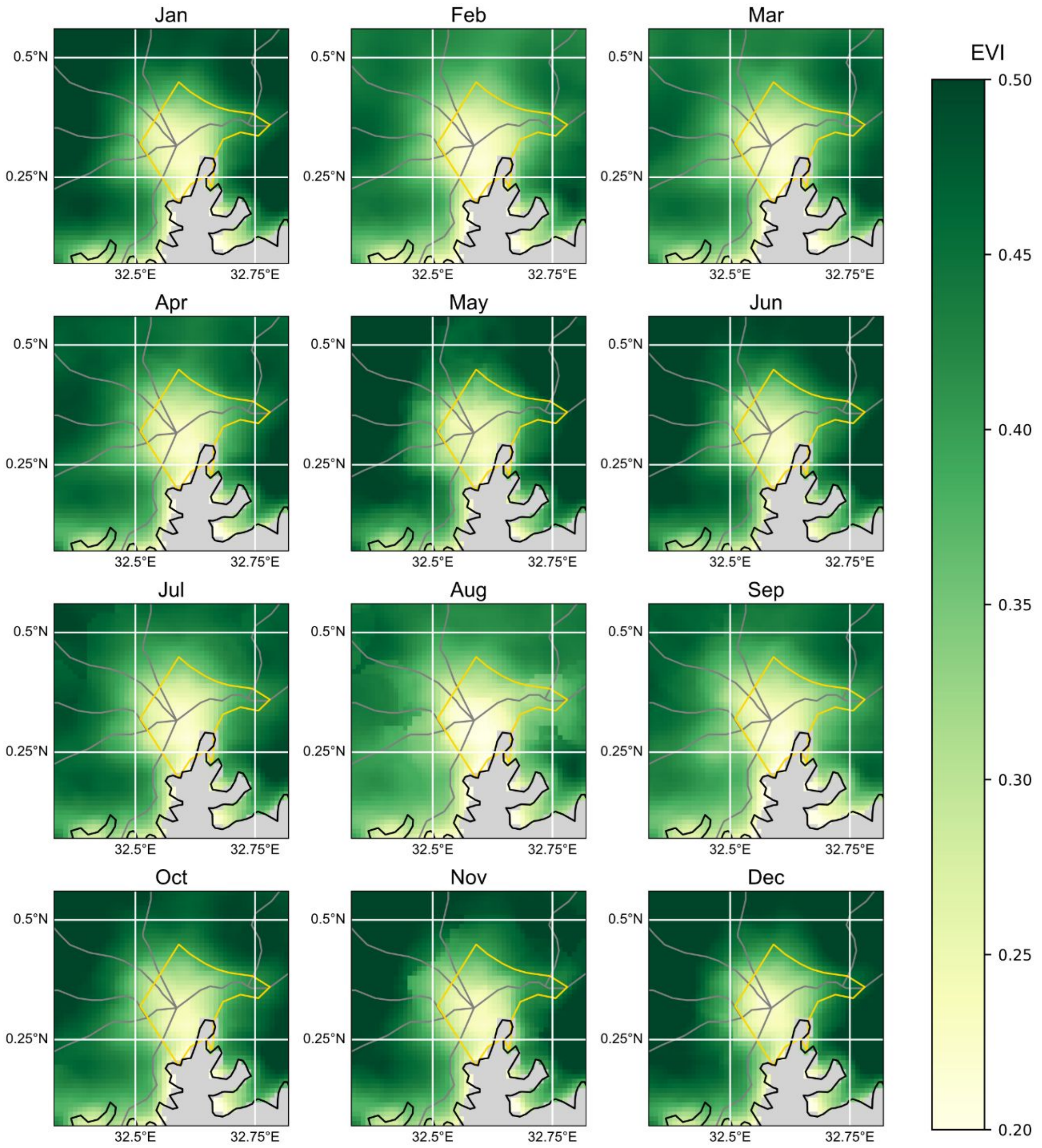

Figure S10. Enhanced vegetation index (EVI) during 2019-2023 aggregated by months at Kampala. The EVI range from 0 to 1 with higher values indicating higher levels of greenery. Note that that colourbar had been rescaled to 0.2 to 0.5 to show greater contrast. Grey areas on the map indicate water areas, grey lines indicate major roads and the yellow outline indicate the urban centre boundary.

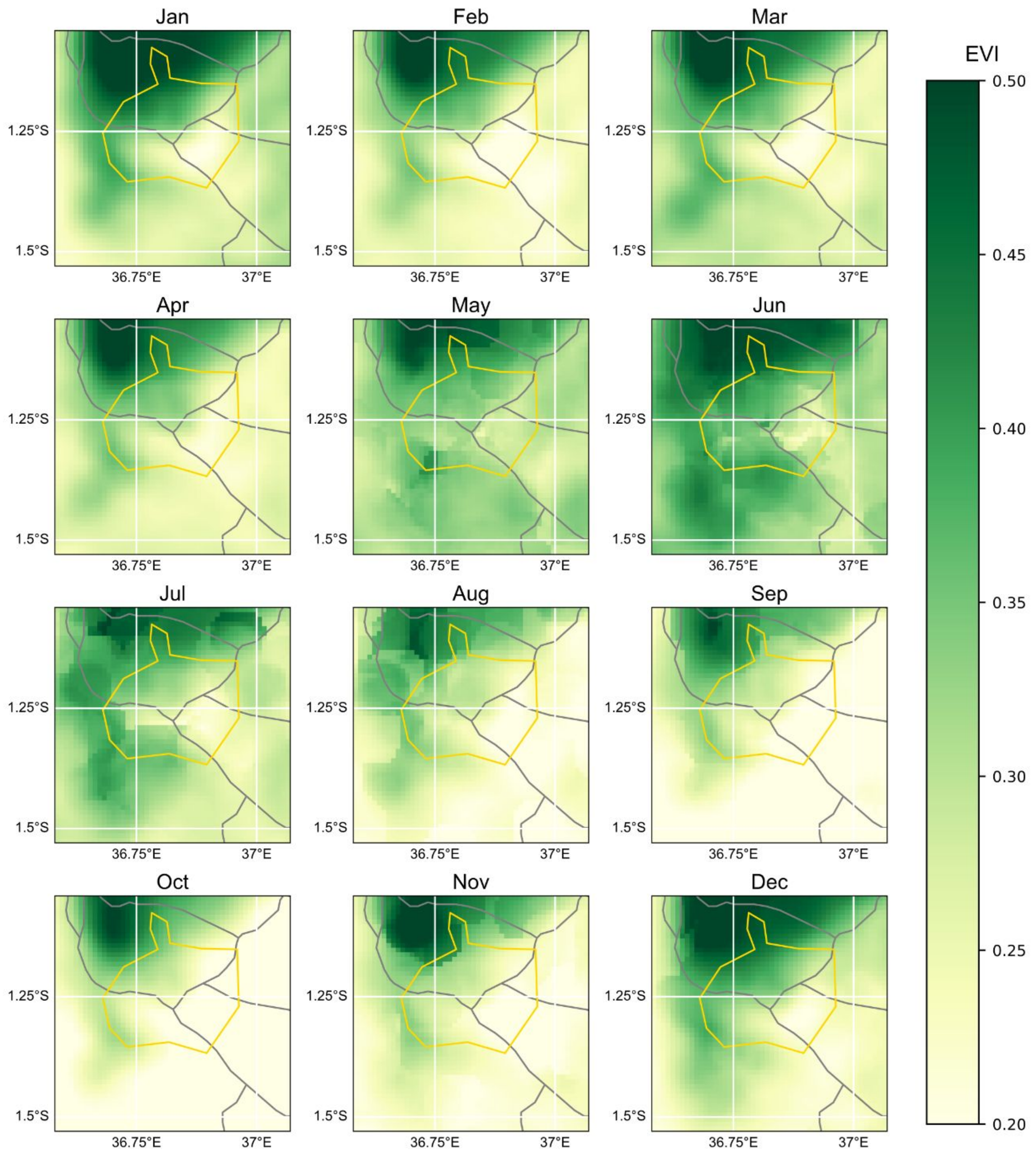

Figure S11. Enhanced vegetation index (EVI) during 2019-2023 aggregated by months at Nairobi. The EVI range from 0 to 1 with higher values indicating higher levels of greenery. Note that that colourbar had been rescaled to 0.2 to 0.5 to show greater contrast. Grey lines indicate major roads and the yellow outline indicate the urban centre boundary.

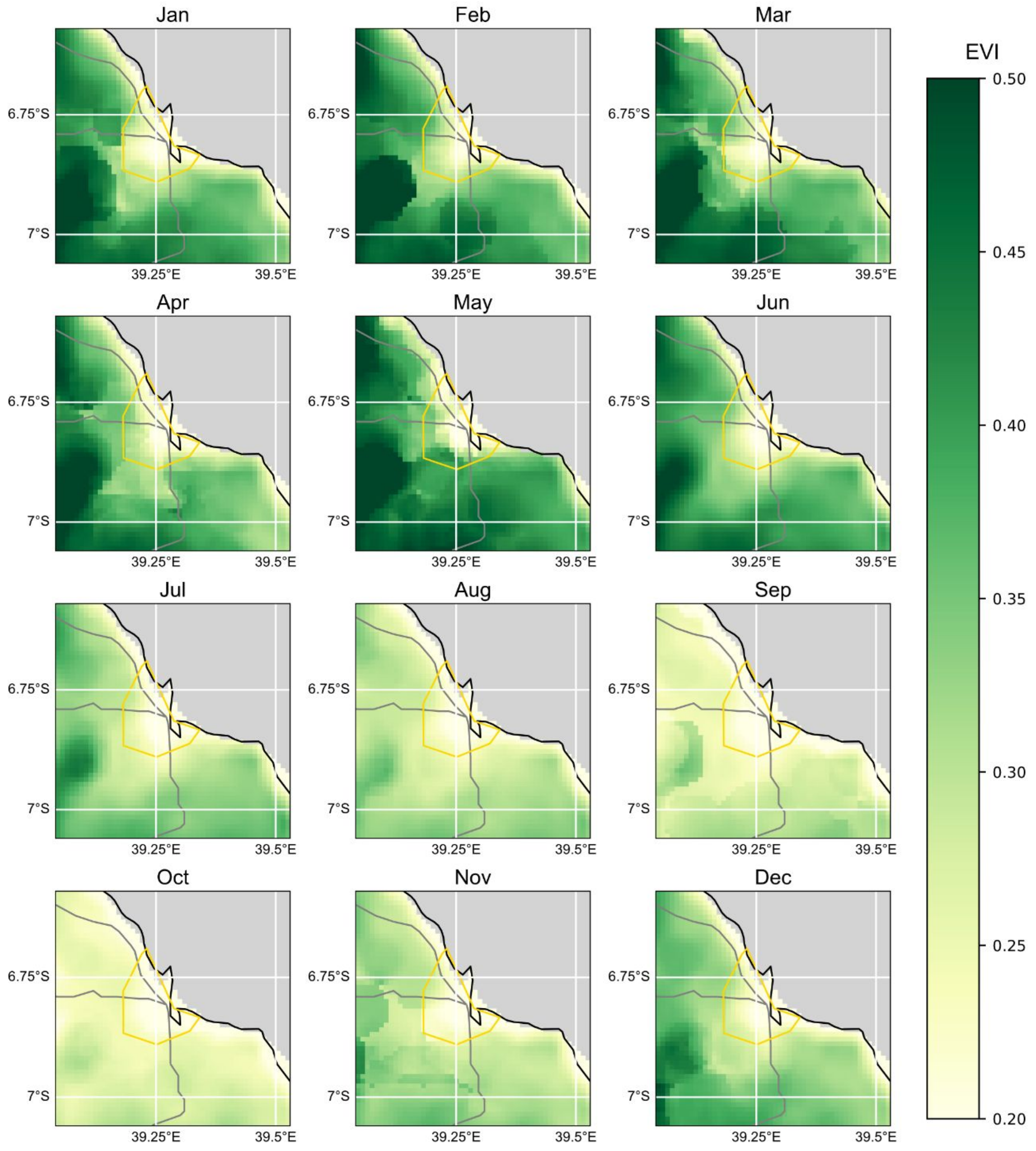

Figure S12. Enhanced vegetation index (EVI) during 2019-2023 aggregated by months at Dar es Salaam. The EVI range from 0 to 1 with higher values indicating higher levels of greenery. Note that that colourbar had been rescaled to 0.2 to 0.5 to show greater contrast. Grey areas on the map indicate water areas, grey lines indicate major roads and the yellow outline indicate the urban centre boundary.

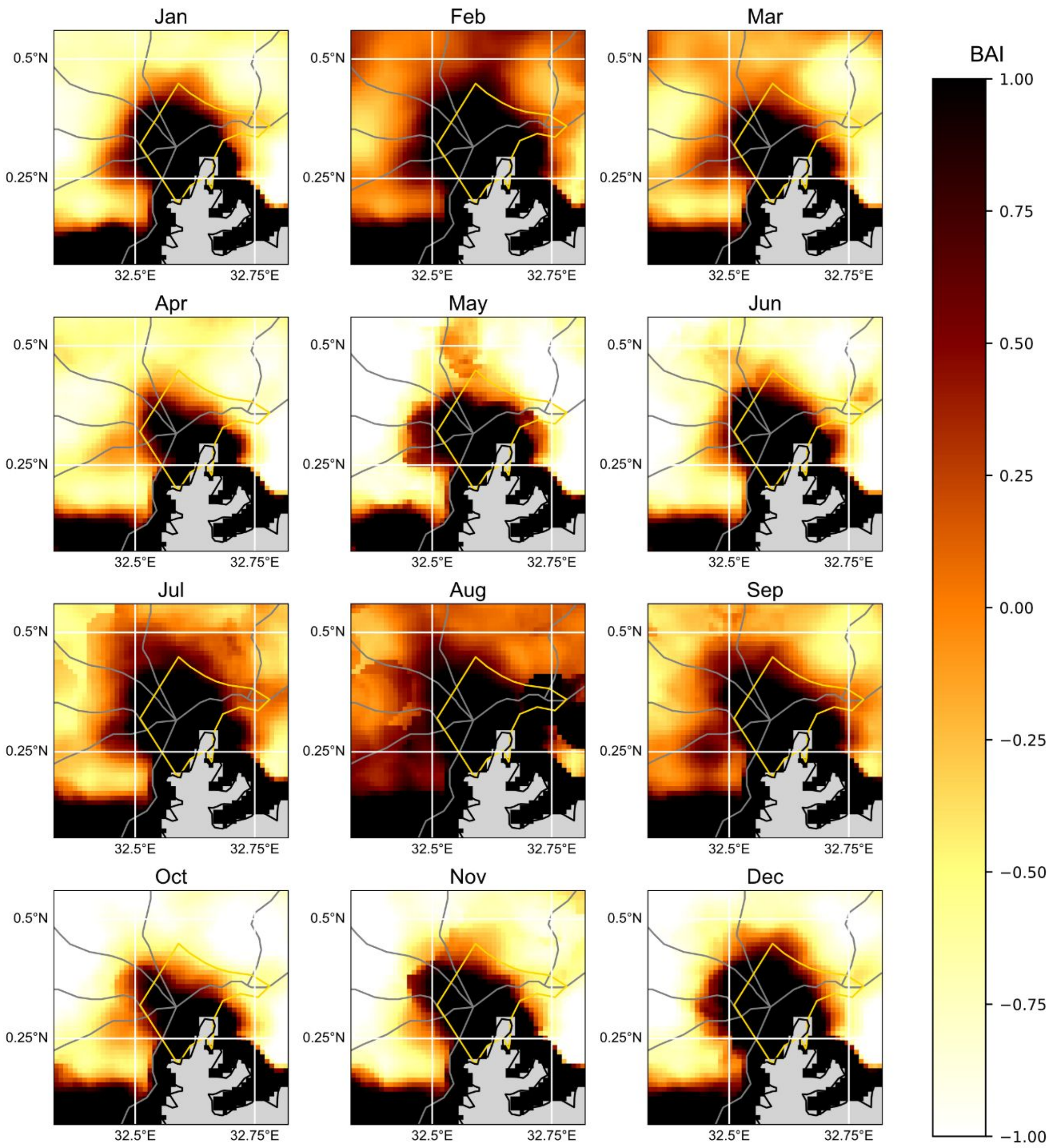

Figure S13. Scaled values of Burn Area Index (BAI) during 2019-2023 aggregated by months at Kampala. Values of 0 indicate mean values, aggregated over entire area from 2019-2023, and ±1 values indicate ±1 standard deviation. Thus, higher values indicate higher severity of burned area. Note the possibility of contamination by water signature (Text S1). Grey areas on the map indicate water areas, grey lines indicate major roads and the yellow outline indicate the urban centre boundary.

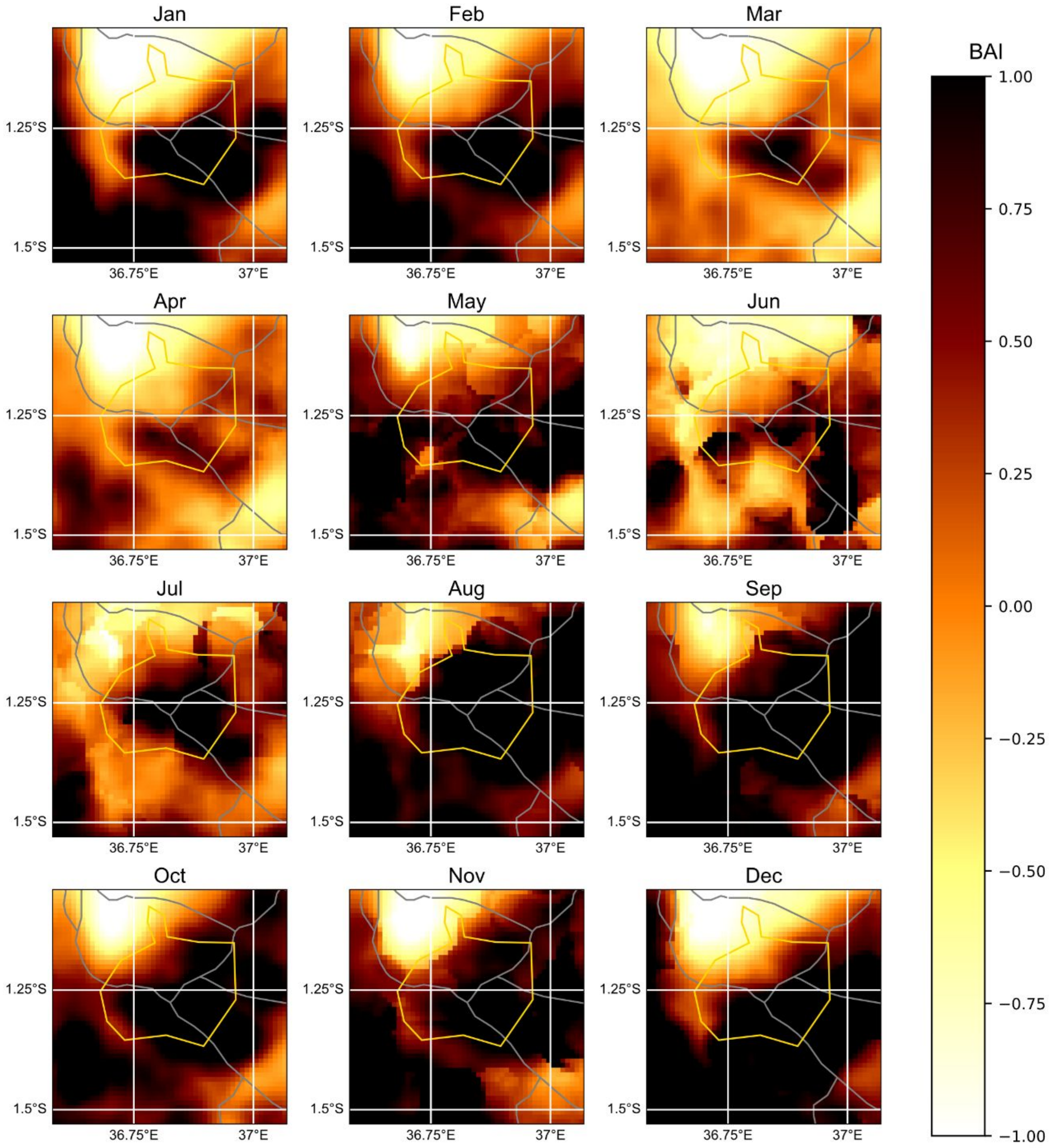

Figure S14. Scaled values of Burn Area Index (BAI) during 2019-2023 aggregated by months at Nairobi. Values of 0 indicate mean values, aggregated over entire area from 2019-2023, and ±1 values indicate ±1 standard deviation. Thus, higher values indicate higher severity of burned area. Note the possibility of contamination by water signature (Text S1). Grey lines indicate major roads and the yellow outline indicate the urban centre boundary.

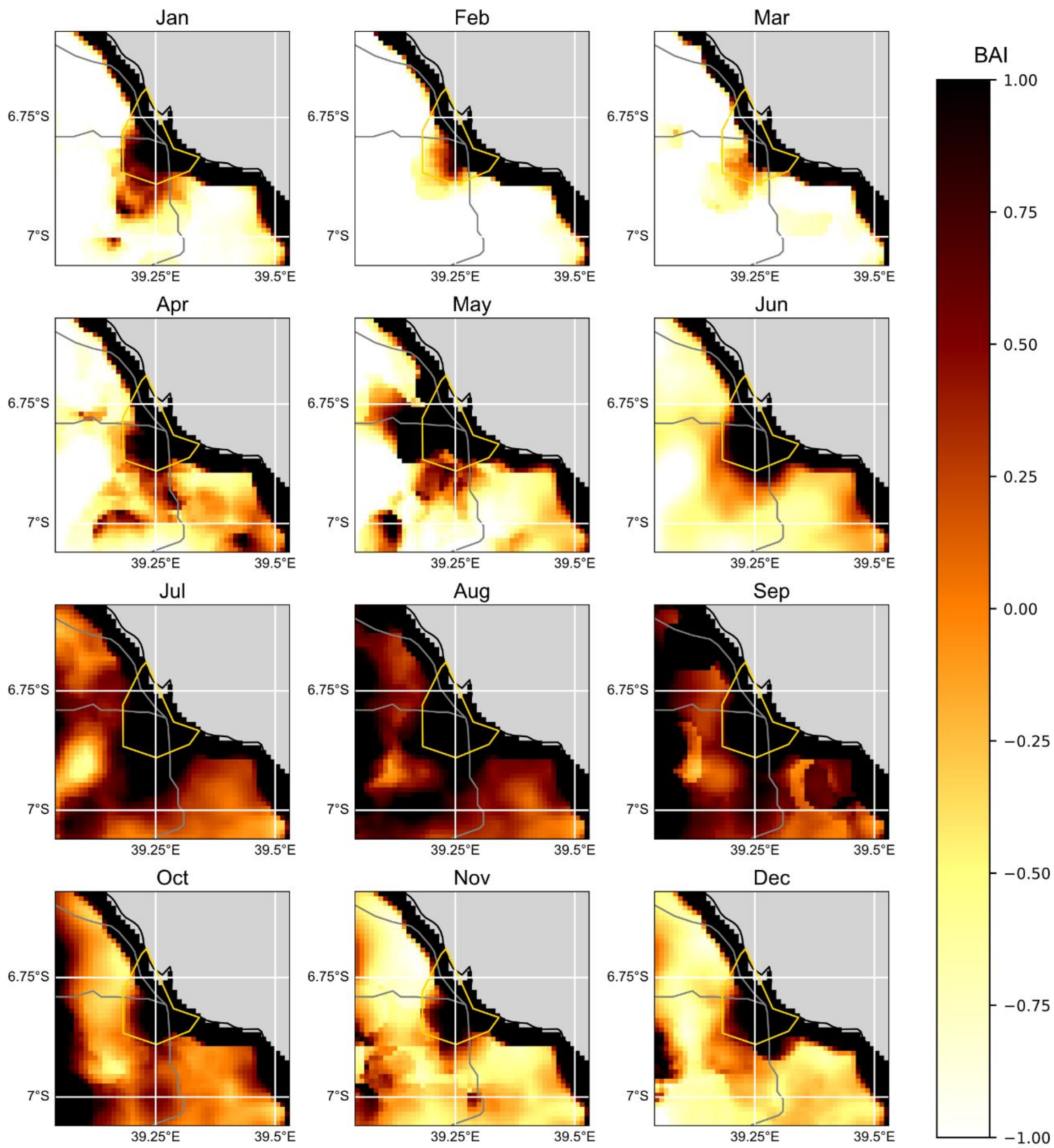

Figure S15. Scaled values of Burn Area Index (BAI) during 2019-2023 aggregated by months at Dar es Salaam. Values of 0 indicate mean values, aggregated over entire area from 2019-2023, and ±1 values indicate ±1 standard deviation. Thus, higher values indicate higher severity of burned area. Note the possibility of contamination by water signature (Text S1). Grey areas on the map indicate water areas, grey lines indicate major roads and the yellow outline indicate the urban centre boundary.

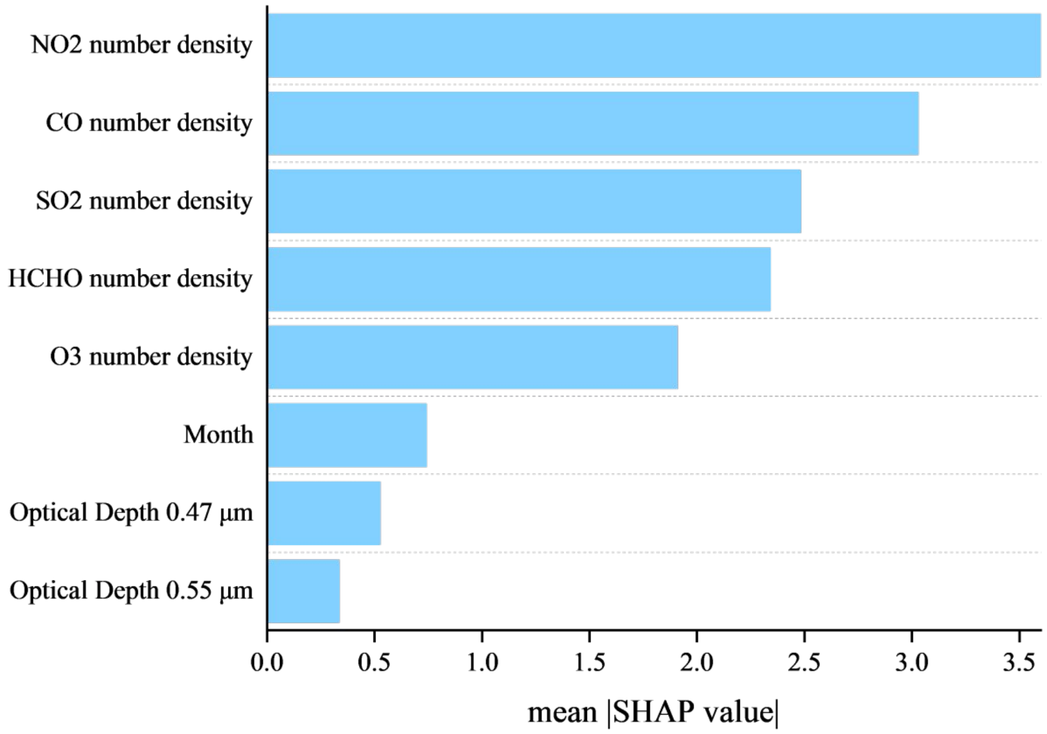

Figure S16. Mean absolute SHAP values of the features used in the XGBoost algorithm. SHAP values are indications of the impact on model output magnitude with higher values implying higher importance.

Table S1. Details of ground-based air quality measurements at the various sites

Site Name	Coordinates		Country	Type
Rubaga, Kampala	0.298°, 32.555°	Urban centre	Uganda	Calibrated data from low-cost sensors (Plantower Sensors PMS 5003)
Bukasa, Kira Municipality	0.299°, 32.615°	Urban centre		
Luwafu, Makindye	0.269°, 32.588°	Urban centre		
Nakawa, KCCA Head Office	0.356°, 32.566°	Urban centre		
Bugolobi, Kampala	0.312°, 32.620°	Urban centre		
Nyendo, Masaka	-0.319°, 31.760°	suburban		
Lower Bugongi, Kabale	-1.241°, 29.988°	suburban		
Kabale Town, Kabale	-1.259°, 29.992°	Urban centre		
Mbarara University, Mbarara	-0.594°, 30.596°	suburban		
Buwenge North, Jinja	0.650°, 33.172°	suburban		
Makerere University, Kampala	0.332°, 32.568°	Urban centre		Reference grade monitor (Met One Beta Attenuation Monitor Model 1022)
University of Nairobi's Parklands Campus, Nairobi	-1.268°, 36.819°	Urban centre	Kenya	Reference grade monitor (Met One Beta Attenuation Monitor Model 1022)
US embassy, Nairobi	-1.234°, 36.812°	Urban centre		Reference grade monitor (Teledyne T640 PM Mass Monitor)

Table S2. Gridded datasets obtained from Google Earth Engine and their corresponding collection name.

Variable	Resolution	Google Earth Engine Collection
NO_2 number density (mol/m^2)	~3.5 km x 5.5 km	COPERNICUS/S5P/NRTI/L3_NO2
SO_2 number density (mol/m^2)		COPERNICUS/S5P/NRTI/L3_SO2
CO number density (mol/m^2)		COPERNICUS/S5P/NRTI/L3_CO
O_3 number density (mol/m^2)		COPERNICUS/S5P/OFFL/L3_O3_TCL
HCHO number density (mol/m^2)		COPERNICUS/S5P/NRTI/L3_HCHO
Optical Depth 0.47 µm<br>Optical Depth 0.55 µm	~1 km x 1 km	MODIS/061/MCD19A2_GRANULES
Enhanced Vegetation Index (EVI)	~0.5 km x 0.5 km	MODIS/MCD43A4_006_EVI
Burn Area Index (BAI)		MODIS/MCD43A4_006_BAI
Temperature (K)<br>Dewpoint temperature (K)<br>u wind component (m/s)<br>v wind component (m/s)	~10 km x 10 km	ECMWF/ERA5_LAND/MONTHLY_AGGR
Rainfall (mm/hr)	~10 km x 10 km	NASA/GPM_L3/IMERG_MONTHLY_V07

Table S3. Spearman's correlation values of meteorological variables with $PM_{2.5}$ concentration. Asterisks indicate statistical significance at p-value<0.05.

	Kampala	Nairobi	Dar es Salaam
Temperature	-0.07	-0.46*	-0.33*
Relative humidity	-0.50*	-0.21	-0.33*
Rainfall	-0.46*	-0.53*	-0.57*
Wind speed	-0.26*	-0.26*	0.60*

Supporting References

(1) Sserunjogi, R.; Ssematimba, J.; Okure, D.; Ogenrwot, D.; Adong, P.; Muyama, L.; Nsimbe, N.; Bbaale, M.; Bainomugisha, E. Seeing the Air in Detail: Hyperlocal Air Quality Dataset Collected from Spatially Distributed AirQo Network. *Data in Brief* **2022**, *44*, 108512. https://doi.org/10.1016/j.dib.2022.108512.

(2) Okure, D.; Ssematimba, J.; Sserunjogi, R.; Gracia, N. L.; Soppelsa, M. E.; Bainomugisha, E. Characterization of Ambient Air Quality in Selected Urban Areas in Uganda Using Low-Cost Sensing and Measurement Technologies. *Environ. Sci. Technol.* **2022**, *56* (6), 3324–3339. https://doi.org/10.1021/acs.est.1c01443.

(3) Adong, P.; Bainomugisha, E.; Okure, D.; Sserunjogi, R. Applying Machine Learning for Large Scale Field Calibration of Low-cost $PM_{2.5}$ and PM_{10} Air Pollution Sensors. *Applied AI Letters* **2022**, *3* (3), e76. https://doi.org/10.1002/ail2.76.

(4) Gobeli, D.; Schloesser, H.; Pottberg, T. Met One Instruments BAM-1020 Beta Attenuation Mass Monitor US-EPA PM2. 5 Federal Equivalent Method Field Test Results. In *The Air & Waste Management Association (A&WMA) Conference, Kansas City, MO*; Citeseer, 2008; Vol. 2.

(5) Lyapustin, A.; Wang, Y.; Korkin, S.; Huang, D. MODIS Collection 6 MAIAC Algorithm. *Atmos. Meas. Tech.* **2018**, *11* (10), 5741–5765. https://doi.org/10.5194/amt-11-5741-2018.

(6) Liu, N.; Zou, B.; Feng, H.; Wang, W.; Tang, Y.; Liang, Y. Evaluation and Comparison of Multiangle Implementation of the Atmospheric Correction Algorithm, Dark Target, and Deep Blue Aerosol Products over China. *Atmos. Chem. Phys.* **2019**, *19* (12), 8243–8268. https://doi.org/10.5194/acp-19-8243-2019.

(7) Mhawish, A.; Banerjee, T.; Sorek-Hamer, M.; Lyapustin, A.; Broday, D. M.; Chatfield, R. Comparison and Evaluation of MODIS Multi-Angle Implementation of Atmospheric Correction (MAIAC) Aerosol Product over South Asia. *Remote Sensing of Environment* **2019**, *224*, 12–28. https://doi.org/10.1016/j.rse.2019.01.033.

(8) Huete, A.; Didan, K.; Miura, T.; Rodriguez, E. P.; Gao, X.; Ferreira, L. G. Overview of the Radiometric and Biophysical Performance of the MODIS Vegetation Indices. *Remote Sensing of Environment* **2002**, *83* (1–2), 195–213. https://doi.org/10.1016/S0034-4257(02)00096-2.

(9) Chuvieco, E.; Martín, M. P.; Palacios, A. Assessment of Different Spectral Indices in the Red-near-Infrared Spectral Domain for Burned Land Discrimination. *International Journal of Remote Sensing* **2002**, *23* (23), 5103–5110. https://doi.org/10.1080/01431160210153129.

(10) Liu, W.; Wang, L.; Zhou, Y.; Wang, S.; Zhu, J.; Wang, F. A Comparison of Forest Fire Burned Area Indices Based on HJ Satellite Data. *Nat Hazards* **2016**, *81* (2), 971–980. https://doi.org/10.1007/s11069-015-2115-x.

(11) Copernicus Climate Change Service. ERA5-Land Monthly Averaged Data from 1950 to Present, 2019. https://doi.org/10.24381/CDS.68D2BB30.

(12) Huffman, G. J.; Stocker, E. F.; Bolvin, D. T.; Nelkin, E. J.; Tan, J. GPM IMERG Final Precipitation L3 1 Month 0.1 Degree x 0.1 Degree V07, 2023. https://doi.org/10.5067/GPM/IMERG/3B-MONTH/07.

(13) Alduchov, O. A.; Eskridge, R. E. Improved Magnus Form Approximation of Saturation Vapor Pressure. *J. Appl. Meteor.* **1996**, *35* (4), 601–609. https://doi.org/10.1175/1520-0450(1996)035<0601:IMFAOS>2.0.CO;2.

(14) Li, J.; Zhang, H.; Chao, C.-Y.; Chien, C.-H.; Wu, C.-Y.; Luo, C. H.; Chen, L.-J.; Biswas, P. Integrating Low-Cost Air Quality Sensor Networks with Fixed and Satellite Monitoring Systems to Study Ground-Level PM2.5. *Atmospheric Environment* **2020**, *223*, 117293. https://doi.org/10.1016/j.atmosenv.2020.117293.

(15) Zaidan, M. A.; Motlagh, N. H.; Fung, P. L.; Khalaf, A. S.; Matsumi, Y.; Ding, A.; Tarkoma, S.; Petaja, T.; Kulmala, M.; Hussein, T. Intelligent Air Pollution Sensors Calibration for Extreme Events and Drifts Monitoring. *IEEE Trans. Ind. Inf.* **2023**, *19* (2), 1366–1379. https://doi.org/10.1109/TII.2022.3151782.

(16) Schubert, E.; Sander, J.; Ester, M.; Kriegel, H. P.; Xu, X. DBSCAN Revisited, Revisited: Why and How You Should (Still) Use DBSCAN. *ACM Trans. Database Syst.* **2017**, *42* (3), 1–21. https://doi.org/10.1145/3068335.

(17) Oguge, O.; Nyamondo, J.; Adera, N.; Okolla, L.; Okoth, B.; Anyango, S.; Afulo, A.; Kumie, A.; Samet, J.; Berhane, K. Fine Particulate Matter Air Pollution and Health Implications for Nairobi, Kenya. *Environmental Epidemiology* **2024**, *8* (3), e307. https://doi.org/10.1097/EE9.0000000000000307.

(18) Singh, A.; Avis, W. R.; Pope, F. D. Visibility as a Proxy for Air Quality in East Africa. *Environ. Res. Lett.* **2020**, *15* (8), 084002. https://doi.org/10.1088/1748-9326/ab8b12.

(19) Chen, T.; Guestrin, C. XGBoost: A Scalable Tree Boosting System. In *Proceedings of the 22nd ACM SIGKDD International Conference on Knowledge Discovery and Data Mining*; ACM: San Francisco California USA, 2016; pp 785–794. https://doi.org/10.1145/2939672.2939785.

(20) Marcilio, W. E.; Eler, D. M. From Explanations to Feature Selection: Assessing SHAP Values as Feature Selection Mechanism. In *2020 33rd SIBGRAPI Conference on Graphics, Patterns and Images (SIBGRAPI)*; IEEE: Recife/Porto de Galinhas, Brazil, 2020; pp 340–347. https://doi.org/10.1109/SIBGRAPI51738.2020.00053.

(21) Yu, S.; Eder, B.; Dennis, R.; Chu, S.; Schwartz, S. E. New Unbiased Symmetric Metrics for Evaluation of Air Quality Models. *Atmospheric Science Letters* **2006**, *7* (1), 26–34. https://doi.org/10.1002/asl.125.

(22) Diez, S.; Lacy, S. E.; Bannan, T. J.; Flynn, M.; Gardiner, T.; Harrison, D.; Marsden, N.; Martin, N. A.; Read, K.; Edwards, P. M. Air Pollution Measurement Errors: Is Your Data Fit for Purpose? *Atmos. Meas. Tech.* **2022**, *15* (13), 4091–4105. https://doi.org/10.5194/amt-15-4091-2022.

(23) Botchkarev, A. Performance Metrics (Error Measures) in Machine Learning Regression, Forecasting and Prognostics: Properties and Typology. **2018**. https://doi.org/10.48550/ARXIV.1809.03006.

(24) Schneider, A.; Friedl, M. A.; Potere, D. A New Map of Global Urban Extent from MODIS Satellite Data. *Environ. Res. Lett.* **2009**, *4* (4), 044003. https://doi.org/10.1088/1748-9326/4/4/044003.
